# Embryonic Stem Cells Modulate the Cancer-Permissive Microenvironment of Human Uveal Melanoma

**DOI:** 10.7150/thno.33139

**Published:** 2019-07-09

**Authors:** Jiahui Liu, Zheqian Huang, Liu Yang, Xiaoran Wang, Shoubi Wang, Chaoyang Li, Ying Liu, Yaqi Cheng, Bowen Wang, Xuan Sang, Xiongjun He, Chenjie Wang, Tengfei Liu, ChengXiu Liu, Lin Jin, Chang Liu, Xiaoran Zhang, Linghua Wang, Zhichong Wang

**Affiliations:** 1 State Key Laboratory of Ophthalmology, Zhongshan Ophthalmic Center, Sun Yat-sen University, Guangzhou 510060, P. R. China.; 2 Department of Ophthalmology, Affiliated Hospital of Qingdao University Medical College, Qingdao 266000, P. R. China; 3 Center for Stem Cell Biology and Tissue Engineering, Key Laboratory for Stem Cells and Tissue Engineering, Ministry of Education, Sun Yat-Sen University, Guangzhou 510275, P. R. China; 4 Department of Genomic Medicine, Division of Cancer Medicine, The University of Texas MD Anderson Cancer Center, Houston, Texas 77030, USA.

**Keywords:** embryonic stem cells, neoplasms, microenvironment, PI3K pathway, uveal melanoma

## Abstract

The currently used anti-cancer therapies work by killing cancer cells but result in adverse effects and resistance to treatment, which accelerates aging and causes damage to normal somatic cells. On one hand, chicken and zebrafish embryos can reprogram cancer cells towards a non-tumorigenic phenotype; however, they cannot be used in the clinical practice. On the other hand, embryonic stem cells (ESCs) mimic the early embryonic microenvironment and are easily available. We investigated the therapeutic efficacy of the ESC microenvironment (ESCMe) in human uveal melanoma *in vitro* and *in vivo*.

**Methods**: Human uveal melanoma C918 cells co-cultured with ESCs were used to measure the levels of mRNA and protein of the phosphoinositide 3-kinase (PI3K) pathway. Cell proliferation, invasiveness, and tumorigenicity of C918 cells were also analyzed. To mimic the tumor microenvironment *in vivo*, we co-cultured C918 cells and normal somatic cells with ESCs in a co-culture system and evaluated the therapeutic potential of ESCMe in both cell types. For an *in vivo* study, a mouse tumor model was used to test the safety and efficacy of the transplanted ESC. Elimination of the transplanted ESCs in mice was carried out by using the ESC-transfected with a thymidine kinase suicidal gene followed by administration of ganciclovir to prevent the formation of teratomas by ESCs.

**Results**: *In vitro* studies confirmed that ESCMe inhibits the proliferation, invasiveness, and tumorigenicity of C918 cells, and the PI3K agonist abolished these effects. ESCMe suppressed the various malignant behaviors of uveal melanoma cells but enhanced the proliferation of normal somatic cells both *in vitro* and *in vivo*. Further, we demonstrated that ESCMe suppressed the PI3K pathway in tumor cells but activated in somatic cells.

**Conclusions**: The ESCMe can effectively suppress the malignant phenotype of uveal melanoma cells and modulate the tumor-promoting aging environment by preventing the senescence of normal cells through the bidirectional regulation of the PI3K signaling. Our results suggest that ESC transplantation can serve as an effective and safe approach for treating cancer without killing cells.

## Introduction

Current anti-cancer therapies, including chemotherapy, radiotherapy, and targeted therapy, are employed to kill the cancer cells. However, such therapies inevitably lead to the development of drug resistance, induce adverse effects, and ultimately cause death to some patients 1, 2, 3. Besides, these therapies can also promote the senescence of normal cells, resulting in a cancer-permissive microenvironment for tumor progression 4,5. According to the adaptive oncogenesis model, the tumor microenvironment is as crucial for the tumor development as are oncogenic mutations 6. In addition to its role in altering selective pressure for oncogenic events, the tumor microenvironment can directly influence the phenotype of malignant cells without altering their genetic makeup 7. Given the pivotal role of microenvironment in cancer initiation, progression, and metastasis, the reversal of the cancer-permissive microenvironment to the cancer-suppressive one may be an ideal approach for the prevention and treatment of various cancers.

A previous study showed that the early embryo microenvironment could cause malignant melanoma cells to revert to a non-tumorigenic phenotype 8. Such findings suggest that the microenvironment can be modulated to reprogram cancer cells without damaging normal cells, thus avoiding the adverse effects caused by the current anti-cancer therapies. However, the reversal of tumor cell to become non-tumorigenic has only been demonstrated in chicken embryos 9, mouse blastocysts 10, and zebrafish embryos 8. Notably, the capacity of the tumor cell to reverse into non-tumor phenotype decreases as the embryo develops and is almost entirely lost after birth 10,11. However, attempts to reprogram tumor cells using adult stem cells have been unsuccessful 12-14. In contrast, because embryonic stem cells (ESCs) are derived from the inner cell mass of blastocysts, they can provide a microenvironment similar to that of an early embryo. Attempts to reprogram tumor cells with ESC-conditioned medium 15 or ESC extracellular matrix 16 have yielded much weaker effects than those observed with early embryos, likely due to the lack of direct interactions between cancer cells and the embryonic microenvironment. These findings indicate that reprogramming tumor cells requires both an early embryonic microenvironment and cell-to-cell interactions.

In a previous study, we used mouse ESCs to establish an embryo-like microenvironment and tested in a murine leukemia model 17. The ESC microenvironment (ESCMe) suppressed leukemic cells and improved the survival rate of mice. However, hematological malignancies account for only approximately 7% of the overall cancer burden worldwide 18. In a hematologic tumor model, ESCs can easily interact with tumor cells; however, whether an embryonic microenvironment can be established in solid tumors and how it is determined remains unclear. To test the potential application of the ESCMe in solid tumors and gain a better understanding of its underlying mechanisms, we conducted systematic and functional experiments both *in vitro* using the C918 human uveal melanoma cell line, and *in vivo* using xenograft mouse models. Our results indicate that the ESCMe has potent anti-tumor activity through suppression of the PI3K signaling pathway, without any adverse effects on the healthy somatic cells.

## Materials and Methods

### Cell cultures

The C918 cell line was purchased from KeyGen Biotechnology Company (Nanjing, China) and cultured in RPMI 1640 medium (Corning, USA) with 10% FBS (Corning) and 1% penicillin-streptomycin (Gibco, Japan). Mouse ESCs and human MSCs were gifts from Professor Andy Peng Xiang. ESCs were cultured in KnockOut Dulbecco's modified Eagle's medium (DMEM; Gibco) with 10% FBS, 0.1 mM non-essential amino acid (Gibco), 1% GlutaMAX media (Gibco), 0.055 mM 2-mercaptoethanol (Gibco), 5×10^5^ units leukemia inhibitory factor (Millipore, USA), and 1% penicillin-streptomycin. The characterization of ESCs can be seen in Figure S1. MSCs were cultured in DMEM (Corning) with 10% FBS, 2% basic fibroblast growth factor (bFGF, Invitrogen, USA), and 1% penicillin-streptomycin. The characterization of ESCs can be seen in Figure S2.The CEC cell line, established in our laboratory previously 19, was cultured in DMEM with 10% FBS, 10 ng/ml human epidermal growth factor (hEGF, Pepro Tech, USA), 5 mg/ml insulin (Sigma, USA), 5 mg/ml human transferrin (Sigma), 0.4 mg/ml hydrocortisone (MB-Chem, India), 2 mM L-glutamine (Gibco), and 1% penicillin-streptomycin. Human RPE cells were isolated from the eyeballs of human donors as described previously 20 and cultured in DMEM/F12 (Corning) with 10% FBS and 1% penicillin- streptomycin. TK-transfected, green fluorescent protein-labeled ESCs were constructed as described previously 17 and grown in ESC culture medium. ESC-CM was collected from cultured ESCs every day, filtered through a 0.22-mm filter (Millex, USA), and preserved at -20 °C.

### Co-culture systems

RPE cells (CM-DiI), C918 cells (DiD), MSCs(Dio) and CECs(Dio) were stained with cell-labeling solution (Invitrogen) according to manufacturer's protocol. For the 2-cell line co-culture studies, 6×10^5^ DiD-labeled C918 cells were plated in a 75-cm^2^ culture flask with 6×10^5^ green fluorescent protein-labeled ESCs, 6×10^5^ DiO -labeled MSCs or CECs. ESCs (8×10^4^ cells/well, placed in the upper chamber) were co-cultured with C918 cells (8×10^4^ cells/well, placed in the lower chamber) in 6-well chambers (0.1 μm) in the TCo system. Culture conditions consisted of C918 culture medium with ESC, MSC, or CEC culture medium at a ratio of 1:1. For control groups, C918 was cultured alone in the corresponding medium. For the 3-cell line co-culture studies, CM-DiI-labeled RPE cells (5,000 cells/cm^2^) and DiD-labeled C918 cells (5,000 cells/cm^2^) were co-cultured with ESCs (5,000 cells/cm^2^) in the CCo system. The control group consisted of CM-DiI-labeled RPE cells (7,500 cells/ cm^2^) and DiD-labeled C918 cells (7,500 cells/cm^2^) in the CCo system. The culture condition was mixed 1:1 by volume with RPE cell culture medium and C918 culture medium. CCo cells were collected after 72 h using fluorescence-activated cell sorting (BD FACSAria Fusion, USA).

### Cell cycle analysis

Cells were fixed with 75% ethanol at -20 °C overnight. Then the cells were stained with 50 mg/ml propidium iodide (BD), incubated with 10 mg/ml RNase A stock solution for 3 h at 4 °C, and assessed on an LSRFortessa flow cytometer (BD). Data were analyzed using Modfit software.

### Apoptosis assay

Staining cells were evaluated with Annexin V-APC/7-aminoactinomycin D (Invitrogen) according to the manufacturer's protocol. The samples were analyzed with a BD LSRFortessa flow cytometer.

### Migration assay

C918 cells were resuspended in serum-free RPMI 1640 medium and seeded onto the upper chambers of Boyden chambers (Corning). RPMI 1640 medium with 10% FBS were then added to the lower chambers. After incubating for 3 h, the adherent cells were stained with a dye solution containing 0.05% crystal violet, and the stained cells in 3 randomly selected high-power fields were counted under a microscope (Leica, Germany).

### Invasion assay

The cells were plated into the upper chamber (BD Matrigel Invasion Chamber, USA) and cultured as described for the migration assay. After 6 h, cells that invaded through the membrane were fixed, stained, photographed, and counted as described for the migration assay.

### Clone formation assay

Cells were seeded into 6-well plates (200 C918 cells/well; 1000 RPE cells/well) and cultured for 7-10 days. Clones were visualized by crystal violet staining and counted.

### Wound-healing assay

C918 cells were plated in 96-well plates. When the cell confluence reached 90-100%, they were scratched with a 10-μl pipette tip and imaged every 3 h using an inverted light microscope (Leica).

### Vascular mimicry (VM) assay

C918 cells were seeded onto Matrigel (BD Biosciences) and incubated for 6 h. Morphological studies were then performed using an inverted light microscope (Leica).

### Cell adhesion assay

The cell adhesion assay was performed as described previously 21. The cell adhesion rate was determined by dividing the number of adherent C918 cells by the number of cells initially seeded and expressed as a percentage.

### CCK-8 cell proliferation assay

C918 cells (200 cells/well) or RPE cells (700 cells/well) were seeded in a 96-well plate and cultured for 24 h. Subsequently, every 24 h, CCK-8 reagent (Dojindo Molecular Technologies, Japan) was added to the cell culture media for 1 h at 37 °C. Absorbance was measured at an optical density of 450 nm in a spectrophotometric plate reader (BioTek, USA).

### RT-qPCR

Total RNA was isolated from cell cultures and tissues using an RNeasy Plus Mini kit (Qiagen, Germany) and RNeasy Fibrous Tissue Mini kit (Qiagen) according to the manufacturer's instructions and then quantified by absorption at 260 nm. cDNA was generated using a PrimeScript™ RT Master Mix (Takara, Japan). Finally, 500 ng of cDNA was used for qPCR. qPCR was performed using a StepOnePlus thermal cycler (ABI, USA) and SYBR® Premix Ex Taq™ (Takara). Relative expression levels were normalized to GAPDH. The PCR primer sequences are listed in Table S1.

### Reagents and antibodies

VO-OHpic was purchased from MedChem Express (New Jersey, USA). The type, source and dilution of antibodies are described in Table S2.

### Western blot analysis

Protein expression in C918 and RPE cells was assessed using Western blotting according to the standard procedure 17. Antibody localization was detected using an enhanced chemiluminescence kit (Amersham, Piscataway, NJ) following the manufacturer's instructions.

### Immunofluorescence assay of cells

C918 and RPE cells were fixed with 4% paraformaldehyde for 20 min after reaching confluence. The cells were permeabilized with 0.1% Triton X-100 (Amresco,USA), and then incubated with the primary antibodies overnight. The cells were then incubated with a secondary antibody for 1 h. Finally, the cells were stained with Hoechst 33342 (Invitrogen), and mounted. The cells were analyzed under an LSM780 or LSM800 confocal microscope (Zeiss, Germany).

### Immunofluorescence assay of tissues

Tumor and skin tissues were fixed with 4% paraformaldehyde; dehydrated with 70%, 80%, 90%, and 95% ethyl alcohol in turn; made transparent with chloroform; and subsequently paraffinized and stored until use. Tissues were cut into 4-μm-thick sections. The sections were heated to 60 °C for 60 min and then washed with xylene, ethyl alcohol, and distilled water in turn. Antigen retrieval was performed in 0.01 mol/L sodium citrate, and the sections were heated 10 more min after steamed. At last, permeabilizing with 0.1% Triton X-100 following the steps described for the immunofluorescence assay of cells.

### *In vivo* tumor experiments

We injected 1×10^6^ C918 cells from control, TCo, or CCo groups subcutaneously into the right flanks of male Balb/c nude mice. Ninety-four days after injection, the mice were euthanized and the tumors were fixed in 4% paraformaldehyde. We randomized mice to receive treatment with ESCs, ESC-CM, or PBS, when the tumor volume reached 150 mm^3^. ESCs (5×10^5^ cells/tumor in 200 μl PBS), ESC-CM (200 μl/tumor), or PBS (200 μl/tumor) was administrated at 2 different sites peritumorally every 7 days. GCV (Sigma, 2 mg/mouse in 200μl PBS) was injected intraperitoneally on day 5 of every treatment cycle. After 3 treatment cycles, the mice were euthanized, and their tumor tissues and surrounding skin tissues, as well as their livers and spleens, were examined.

### Histological analyses

Tissues were mounted onto slides for hematoxylin and eosin (H&E) staining. Slides were imaged on a Pannoramic Digital Slide Scanner (3DHISTECH, Hungary). The slides were stained with CD34-PAS according to the standard procedure. TUNEL labeling was performed following the manufacturer's (KeyGen's) instructions.

### *In vivo* imaging

ESCs were stained with DiR (Invitrogen) and then immediately injected into mice. The mice were putted in an MS FX PRO Imaging System (Bruker, USA). The isoflurane level was set at 1%-2% until complete image acquisition.

### Statistical analyses

GraphPad Prism software was used to perform the statistical analyses. Kaplan-Meier survival plots were generated using a log-rank test (Mantel-Cox test). A 2-tailed unpaired Student t-test was used for analyses comparing only 2 groups, and analysis of variance and an appropriate post hoc test were used for analyses comparing more than 2 groups. All experiments were repeated as indicated. All data are expressed as means ± standard errors of the means (SEMs). P values < 0.05 were considered significant.

## Results

### The ESCMe inhibits the proliferation, invasiveness, and tumorigenicity of C918 cells

**Figure 1:**
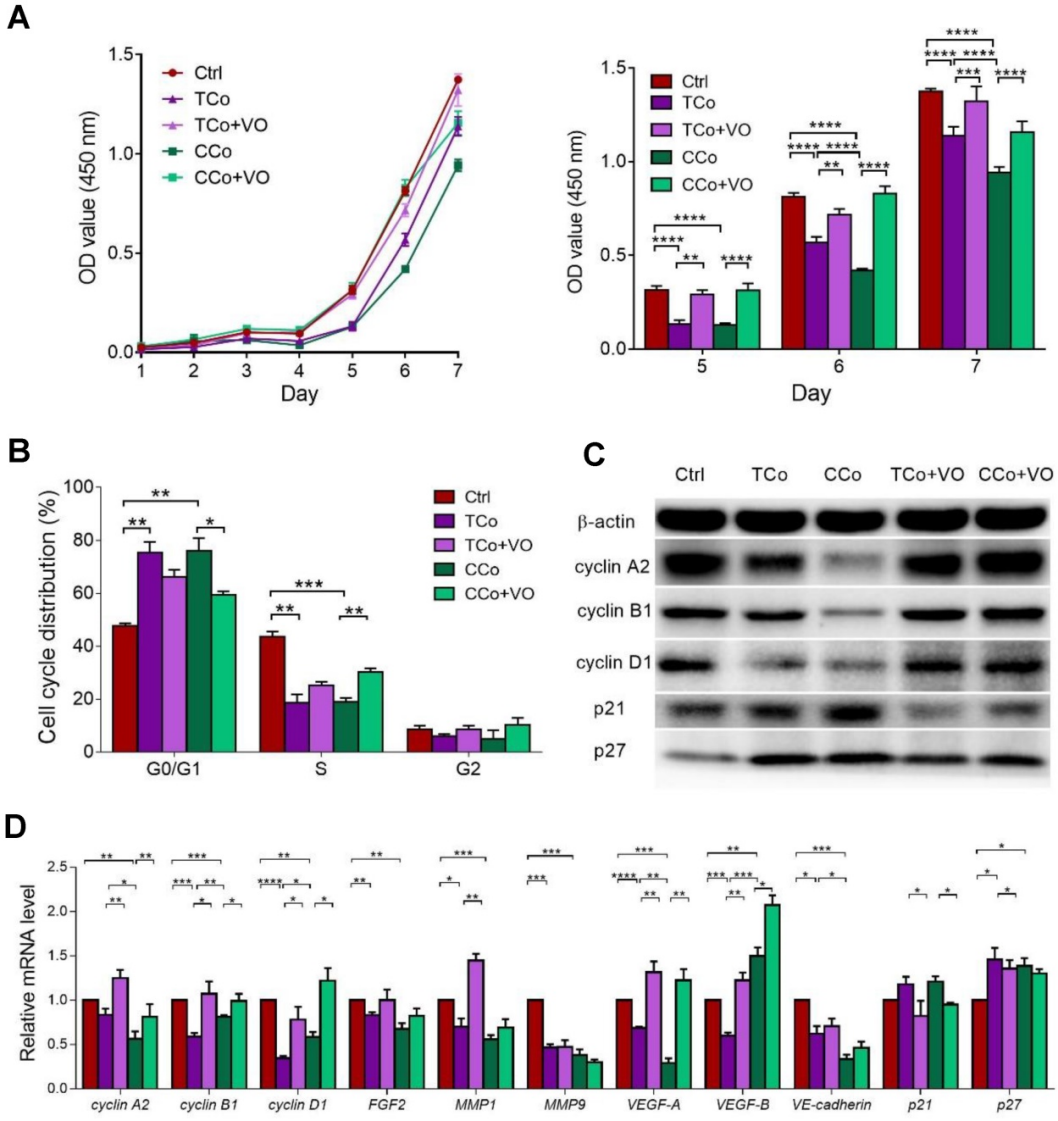
**The ESCMe inhibits the proliferation of C918 cells. (A)** Proliferation of C918 cells sorted from the control (Ctrl), TCo, TCo+VO, CCo, and CCo+VO groups, as assessed by a CCK8 proliferation assay (n = 4 biological repeats). **(B)** Proportion of cell cycle distribution in C918 cells, as assessed by flow cytometry (n = 3 biological repeats). **(C)** Western blotting of cyclin proteins and p21, p27 in C918 cells. β-actin served as the internal control. **(D)** Expression of the cell cycle and metastasis-related markers in C918 cells, as assessed by RT-qPCR. Delta CT means of genes in C918 cells were shown in Table S3.

**Figure 2:**
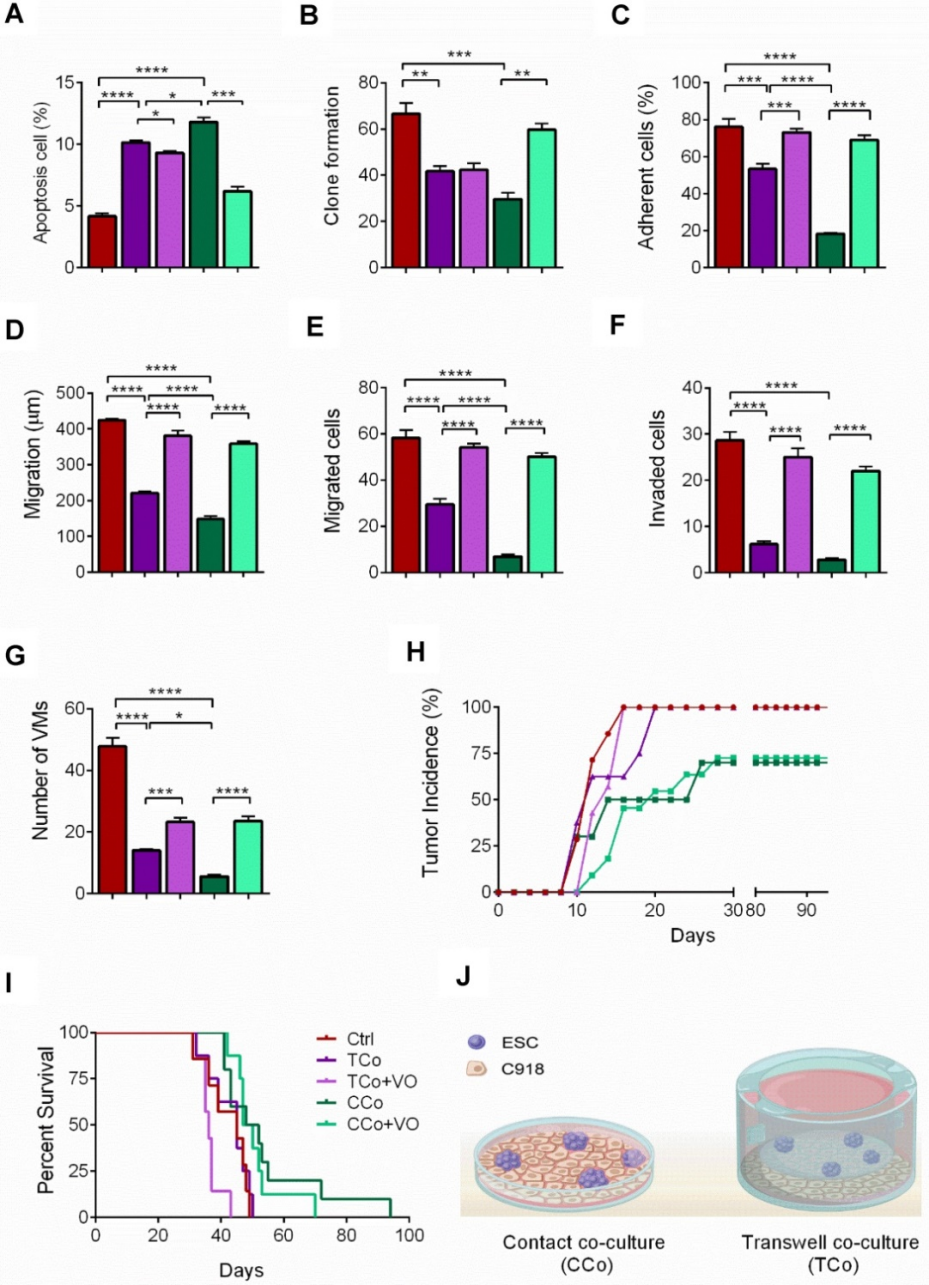
** The ESCMe inhibits the metastasis and tumorigenesis of C918 cells. (A)** Percentages of apoptotic C918 cells (n = 3 biological repeats). **(B)** Clone formation of C918 cells (n = 3 biological repeats). **(C)** Percentages of adherent C918 cells (n = 6 biological repeats). **(D)** Numbers of invaded C918 cells (n = 5 biological repeats). **(E)** Numbers of migrated C918 cells (n = 5 biological repeats). **(F)** Migration distances of C918 cells after 9 h of culture (n = 5 biological repeats). **(G)** Numbers of VMs of C918 cells (n = 5 biological repeats). **(H)** Tumor incidence in mice. **(I)** Kaplan-Meier survival curves for mice (n = 7-10 mice per treatment group). **(J)** Model for the two different co-culture systems. Data are means ± SEMs. *P< 0.05; **P< 0.01; ***P< 0.001; ****P< 0.0001.

To investigate the therapeutic potential of ESCs on human uveal melanoma, C918 cells were co- cultured with ESCs in a cell-to-cell contact co-culture system (CCo group) for 72 h. In the control group, C918 cells were grown alone. Compared with the control group, C918 cells in the CCo group had a significantly reduced cell proliferation (Figure 1A) and a significantly reduced proportion of cells entering the replication S phase (Figure 1B). Consistently, the C918 cells in the CCo group had much lower expression levels of cell cycle proteins, including cyclin A2, cyclin B1, and cyclin D1, and higher expression levels of the cell cycle negative regulatory factors, p21 and p27 (Figure 1C-D). Concomitantly, the C918 cells in the CCo group had higher apoptosis when compared with the control group (Figure 2A, and Figure S3A). In addition, the clone formation rate of the C918 cells in the CCo group was nearly 50% of that in the control group (Figure 2B, and Figure S3B). Taken together, these results suggest that ESCs, through their direct interaction with C918 cells, significantly suppress the proliferation and clone formation of C918 cells and promote their apoptosis.

Next, we examined the effects of ESCs on tumor cell adhesion, invasion, and migration, which are associated with enhanced metastatic capacity. Compared with those in the control group, the C918 cells in the CCo group showed decreased adhesion, migration, and invasion capacities (Figure 2C-F, and Figure S3C-E). VM, a prognostic indicator for a highly invasive behavior 22, as well as tubular network and regular channel formation in the 3D matrix, was exclusively present in the control group, but not in the poorly invasive CCo group (Figure 2G, and Figure S3F). Consistently, the expression levels of metastasis-related effector molecules, including VE-cadherin, VEGF-A, FGF2, and MMPs, were dramatically decreased (Figure 1D, Figure S3G and Figure S4A), whereas the metastasis suppressors, BRMS1 and TXNIP, were upregulated (Figure S4B-C) in the CCo group. Collectively, these data suggest that ESCs significantly inhibit the metastatic capacity of C918 cells.

C918 cells from the CCo and control groups were subcutaneously injected into nude mice. Sixteen days after injection, the overall tumor formation rate was 100% in mice injected with the control group, but was only 50% with the CCo group (Figure 2H). For over three months, the CCo group had a maximum tumor formation rate of 70%. The median survival rate of the mice in the CCo group was higher than that of the mice in the control group (Figure 2I). A TUNEL assay revealed that the CCo group had a higher number of the apoptotic cells when compared with the control group (Figure S4D). The CD34-PAS double staining was used to distinguish VM and normal endothelial- dependent vessels in tumor tissue 23 and revealed that the endothelium-lined vessels and VM channels were fewer in the CCo group than those in the control group (S4E). A histopathological assay revealed a lower nuclear-to-cytoplasmic (N:C) ratio in the CCo group than the control group (Figure S4F), indicating that tumors arising from C918 cells co-cultured with ESCs were less aggressive than those arising from C918 cells cultured alone. Taken together, these results demonstrate that ESCs decreased the malignant activity of C918 cells.

To determine whether direct cell-to-cell contact between ESCs and C918 cells is essential for the reversal of the C918 cell malignant phenotype, we cultured the C918 cells in a transwell (non cell-to-cell contact) co-culture system (TCo group), where C918 cells were placed in the bottom well, and ESCs in the top insert (Figure 2J) and only media were exchanged between them. The proliferation, invasiveness, and tumorigenicity of the C918 cells in the TCo group were suppressed; however, to a significantly lesser extent than those of the C918 cells in the CCo group. These results suggest the importance of cell-to-cell direct contact between ESCs and C918 cells in exerting an anti-tumor activity of the embryonic microenvironment (Figure 1-2 and Figure S3-S4).

### Mesenchymal stem cells (MSCs) and corneal epithelial cells (CECs) cannot suppress the malignant phenotype of C918 cells

**Figure 3:**
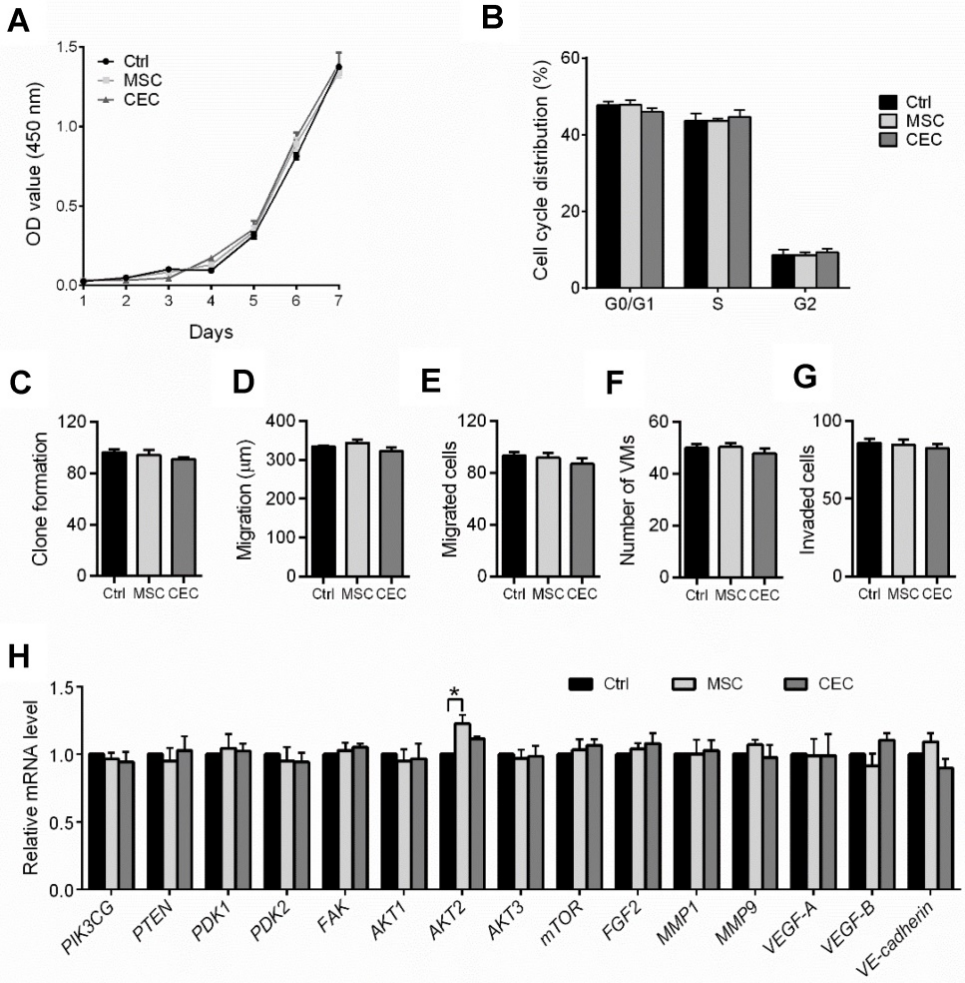
** The ability of tumor reversal of ESCs is attributed to its embryonic properties. (A)** Proliferation of C918 cells sorted from control cultures (Ctrl), MSC co-cultures, and CEC co-cultures, as assessed with a CCK8 proliferation assay (n = 3 biological repeats). **(B)** Cell cycle distribution of C918 cells sorted from Ctrl cultures, MSC co-cultures, and CEC co-cultures, as assessed by flow cytometry (n = 3 biological repeats). **(C)** Clone formation of C918 cells (n = 3 biological repeats). **(D)** The migration distance of C918 cells after 9 h of culture alone (Ctrl), with MSCs, and with CECs (n = 5 biological repeats). **(E)** Numbers of migrated C918 cells (n = 5 biological repeats). **(F)** Numbers of invaded C918 cells (n = 5 biological repeats). **(G)** Numbers of VMs formed by C918 cells (n = 5 biological repeats). **(H)** Expression of metastasis markers and PI3K pathway genes in C918 cells, as assessed by RT-qPCR (n = 3 biological repeats). Data are means ± SEMs. *P< 0.05.

Earlier reports have revealed that the effect of the embryonic microenvironment on tumor reversal is the strongest in the early stages of the embryo but gradually diminishes as the embryo ages 10,11. To confirm this, we cultured C918 cells with human mesenchymal stem cells or human corneal epithelial cells in direct-contact co-culture systems. Neither the malignant phenotype of the C918 cells nor their gene expression levels were significantly changed when the cells were co-cultured with MSCs or CECs (Figure 3). Unlike ESCs, both MSCs and CECs failed to suppress tumor growth and invasiveness, which suggests that non-embryonic cells with multipotent stem cell property cannot reverse the malignant phenotype of the C918 cells.

### The ESCMe reverses tumor malignancy by inhibiting the PI3K pathway

**Figure 4:**
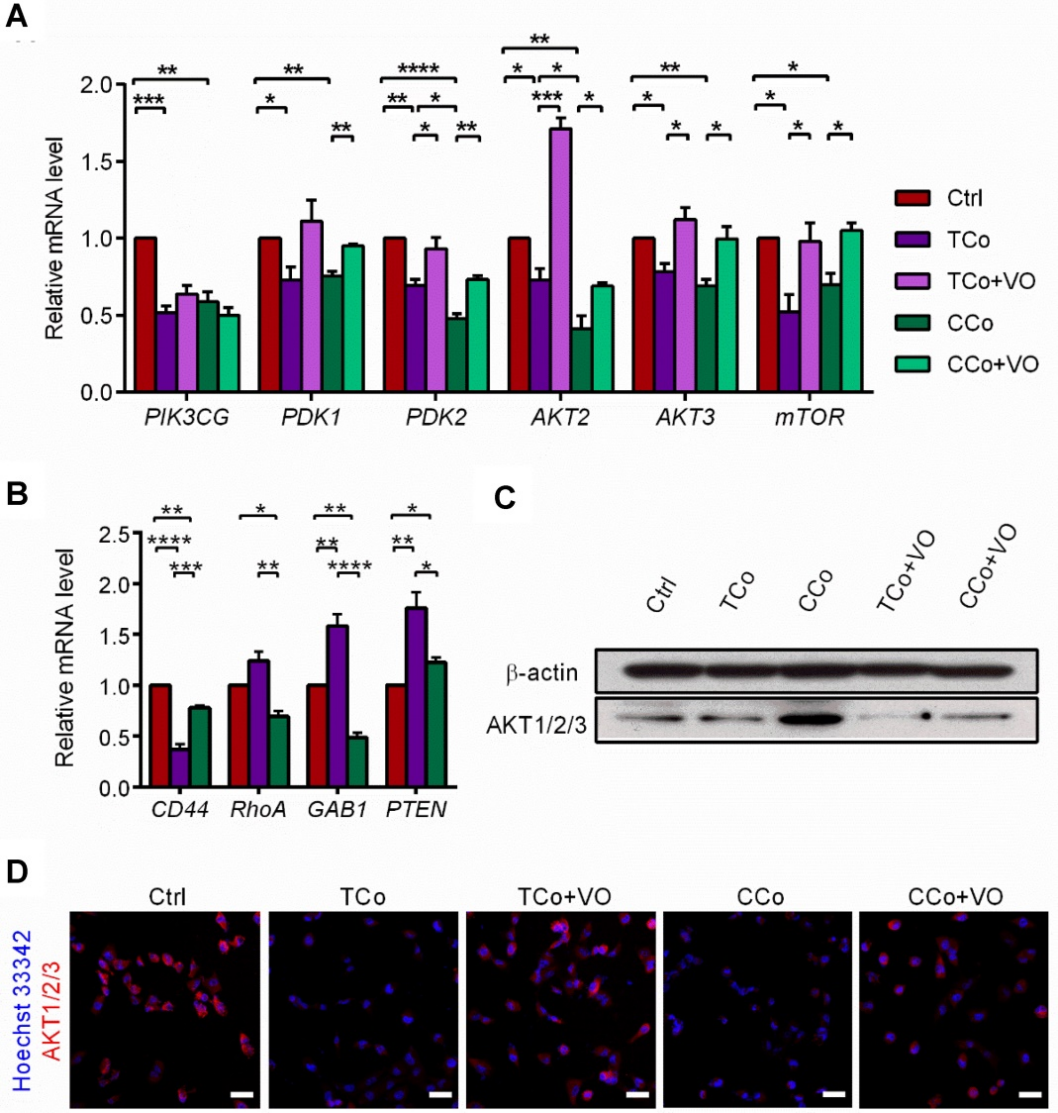
** The ESCMe reverses tumor phenotype by inhibiting the PI3K pathway. (A)** Expression of PI3K pathway genes in C918 cells from the Ctrl, TCo, TCo +VO, CCo, and CCo+VO groups, as assessed by RT-qPCR (n = 3 biological repeats). Delta CT means of genes in C918 cells were shown in Table S3. **(B)** Expression of upstream activators and suppressors of the PI3K pathway in C918 cells from the Ctrl, TCo, and CCo groups, as assessed by RT-qPCR (n = 3 biological repeats). Delta CT means of genes in C918 cells were shown in Table S4. **(C)** Western blotting of AKT1/2/3 in C918 cells from the Ctrl, TCo, TCo +VO, CCo, and CCo+VO groups. β-actin served as the internal control. **(D)** Immunofluorescence assays of AKT1/2/3 in C918 cells. Scale bar, 50 μm. Data are means ± SEMs. *P< 0.05; **P< 0.01; ***P< 0.001; ****P< 0.0001.

The PI3K/AKT pathway promotes tumor development and progression, especially in uveal melanoma 24,25. To understand the mechanisms underlying the reversal of the C918 cells' malignant phenotype, we performed a quantitative gene expression analysis of the key PI3K pathway genes, including *PIK3CG*, *PDK2*, *AKT2*, *AKT3*, and *mTOR.* Expression levels of these genes significantly decreased in the C918 cells from the CCo group, compared with those from the control group (Figure 4A). Additionally, expression of the upstream activators of the PI3K pathway, such as *CD44* and *Gab1* genes, were downregulated in the C918 cells from the CCo group (Figure 4B). On the contrary, expression of the PI3K pathway suppressors, including *PTEN*, *TXNIP,* and *BRMS1* genes, were markedly upregulated in the CCo group when compared with those in the control group (Figure 4B and Figure S4B-C). Our results also indicate that the expression of nearly all these genes was significantly altered in the CCo group than the TCo group (Figure 3A-B and Figure S4B-C), indicating that the direct cell-cell contact approach with ESCs is more effective than the non-contact approach in suppressing the PI3K pathway. To determine whether PI3K pathway activity is necessary for the tumor- suppressing effect of the ESCMe, we treated the co-cultures with the PI3K agonist, VO-OHpic (VO), to stimulate the PI3K signaling (Figure 4C- D). Treatment of VO abolished the anti- cancer effect of the ESCMe on the C918 cells in the two co-culture systems to various extents (Figure 1, Figure 2A-I and Figure S3-S4A). However, the tumor formation capacity of the CCo group remained unchanged upon VO treatment (Figure 2H). These results demonstrate that the ESCMe exerts its anti-tumor effect by inhibiting the PI3K pathway.

### ESCMe suppresses tumor cells while preventing the senescence of normal cells

**Figure 5:**
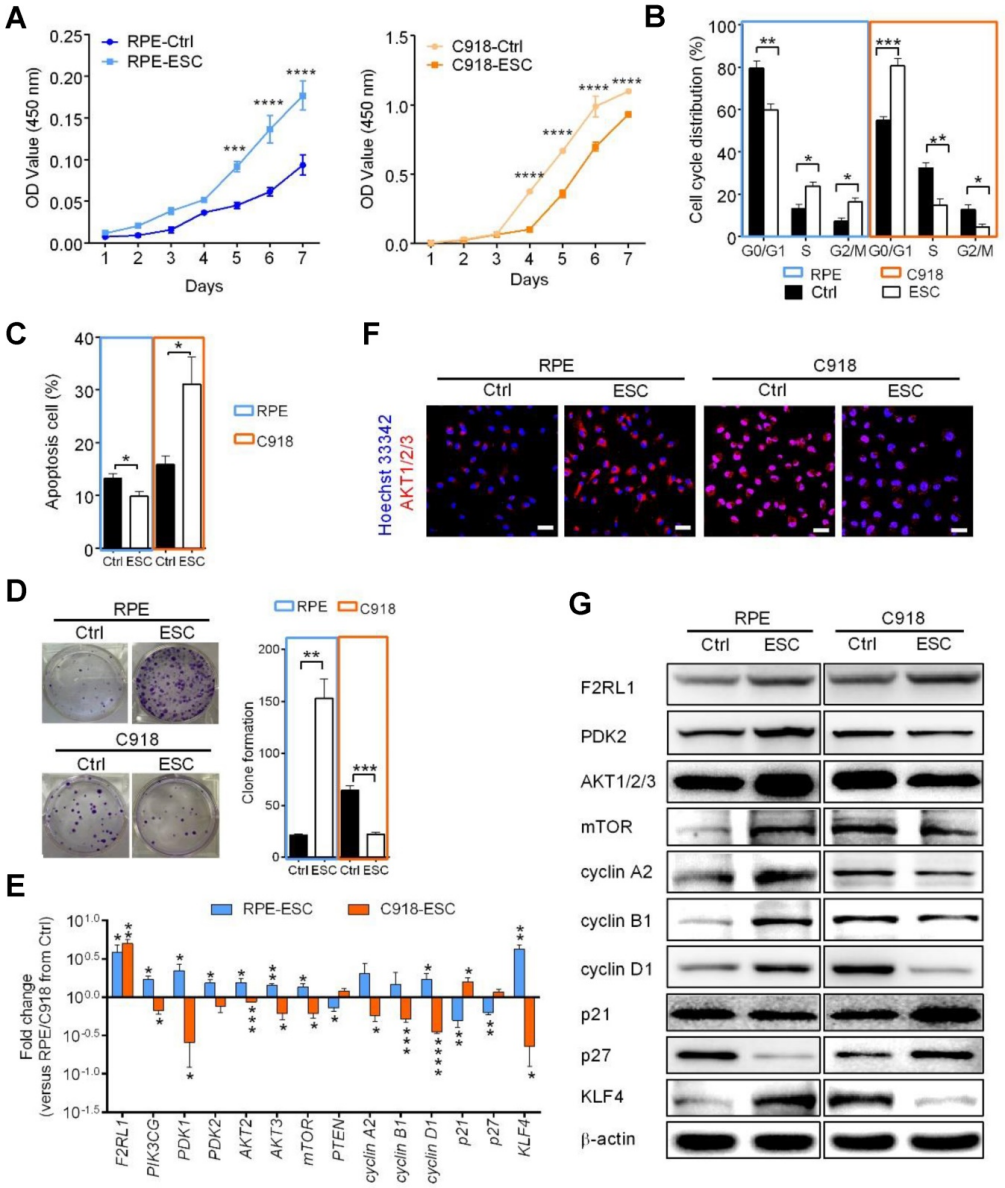
** The ESCMe can suppress the aggressive phenotype of tumor cells while preventing the senescence of normal somatic cells *in vitro*. (A)** Proliferation of RPE and C918 cells sorted from C918 cells co-cultured with RPE cells (Ctrl) and from C918 cells co-cultured with RPE cells and ESCs (ESC), as assessed by CCK8 proliferation assay (n = 4 biological repeats). **(B)** Cell cycle distribution of RPE and C918 cells sorted from the Ctrl and ESC groups, as assessed by flow cytometry (n = 4 biological repeats). **(C)** Percentages of apoptotic RPE and C918 cells sorted from the Ctrl and ESC groups, as assessed by flow cytometry (n = 3 biological repeats). **(D)** Numbers and representative images of clones formed by RPE and C918 cells (n = 3 biological repeats). Delta CT means of genes in RPE and C918 cells were shown in Table S5. **(E)** Fold change of RNA expression in RPE and C918 cells from the ESC group compared with those in the Ctrl group (n = 3 biological repeats). **(F)** Immunofluorescence assays of AKT1/2/3 in RPE and C918 cells from the Ctrl and ESC groups. Scale bar, 50 μm. **(G)** Western blotting of RPE and C918 cells from the Ctrl and ESC groups. β-actin served as the internal control. Data are means ± SEMs. *P< 0.05; **P< 0.01; ***P< 0.001; ****P< 0.0001.

To investigate whether ESC treatment damages normal cells, we cultured human retinal pigment epithelial (RPE) cells and C918 cells together with ESCs in a contact co-culture system to mimic a tumor microenvironment. We observed that ESCs were able to continue suppressing C918 cell proliferation through the inhibition of the PI3K-AKT pathway (Figure 5 and Figure S5-S6). However, compared with the RPE cells in the control group, the ESC-treated RPE cells had a higher proliferation rate (Figure 5A), a faster cell cycle turnaround time (Figure 5B), a decreased apoptosis (Figure 5C), and an increased clone formation ability (Figure 5D). Besides, higher expression levels of the cell cycle effectors, cyclin A2, cyclin B1, and cyclin D1 were observed in the ESC-treated RPE cells (Figure 5E and G, and Figure S5).

Furthermore, ESC treatment prevented the expression of the senescence markers, p21 and p27, at both transcriptional and translational levels (Figure 5E and G, and Figure S5). Since stem cell exhaustion is likely one of the ultimate causes of aging 26, we assessed the expression of KLF4, a marker associated with early stem cells and reprogramming 27,28. Quantitative gene expression and western blot analysis revealed that KLF4 was barely detectable in the RPE cells in the control group, but was markedly upregulated in ESC-treated RPE cells (Figure 5E and G), indicating that ESCs enhanced the stem cell phenotype of RPE cells. Moreover, in contrast to ESC-treated C918 cells, ESC-treated RPE cells had higher expression levels of the PI3K-AKT pathway related-genes (Figure 5E-G, and Figure S6). These results were consistent with our earlier study showing that ESCs could significantly promote the proliferation of terminal cells by stimulating the expression of the markers of some precursor cells 29.

The F2R-like trypsin receptor 1 (F2RL1) (also known as PAR2), a protease-activated receptor expressed on the cell surface of various tissue types, has been shown to play a regulatory role in PI3K signaling and contribute to a broad range of normal and disease-related processes, including embryogenesis, inflammation, and cancer 30-32. Compared with the cells in the control group, the C918 cells and RPE cells co-cultured with ESCs showed an increased expression of F2RL1 (Figure 5E and G). Thus, we speculated that F2RL1 is involved in the cross-talk between ESCs and the *F2RL1*-expressing cells including, cancer cells and normal cells.

**Figure 6:**
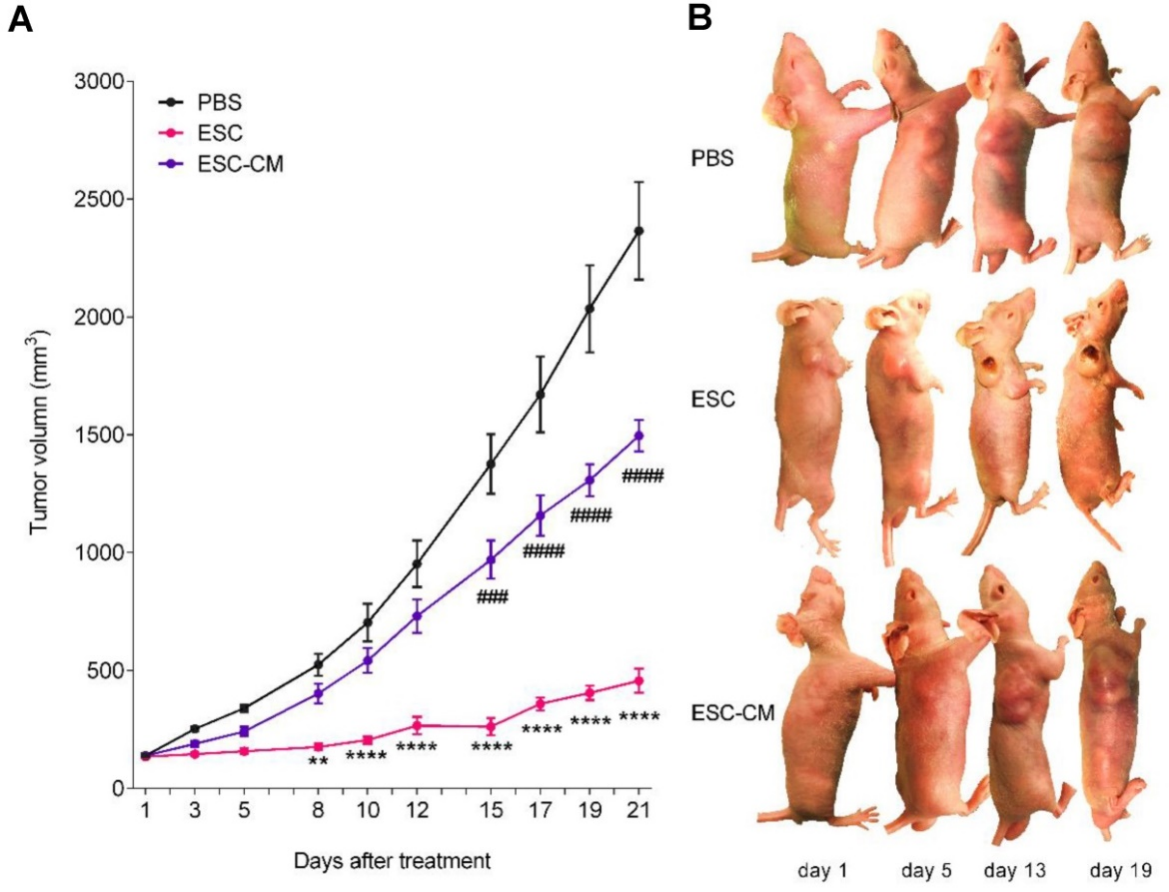
** The ESCMe inhibited tumor growth in xenograft mice models. (A)** Volumes of tumors treated with PBS, ESCs, and ESC-CM, respectively, *in vivo* (n = 8 mice per treatment group). **(B)** Representative images of tumors in mice after treatment. Data are means ± SEMs. * means significant statistical differences between PBS group and ESC group. *P< 0.05; **P< 0.01; ***P< 0.001; ****P< 0.0001. # means significant statistical differences between PBS group and ESC-CM group. #P< 0.05; ##P< 0.01; ###P< 0.001; ####P< 0.0001.

**Figure 7:**
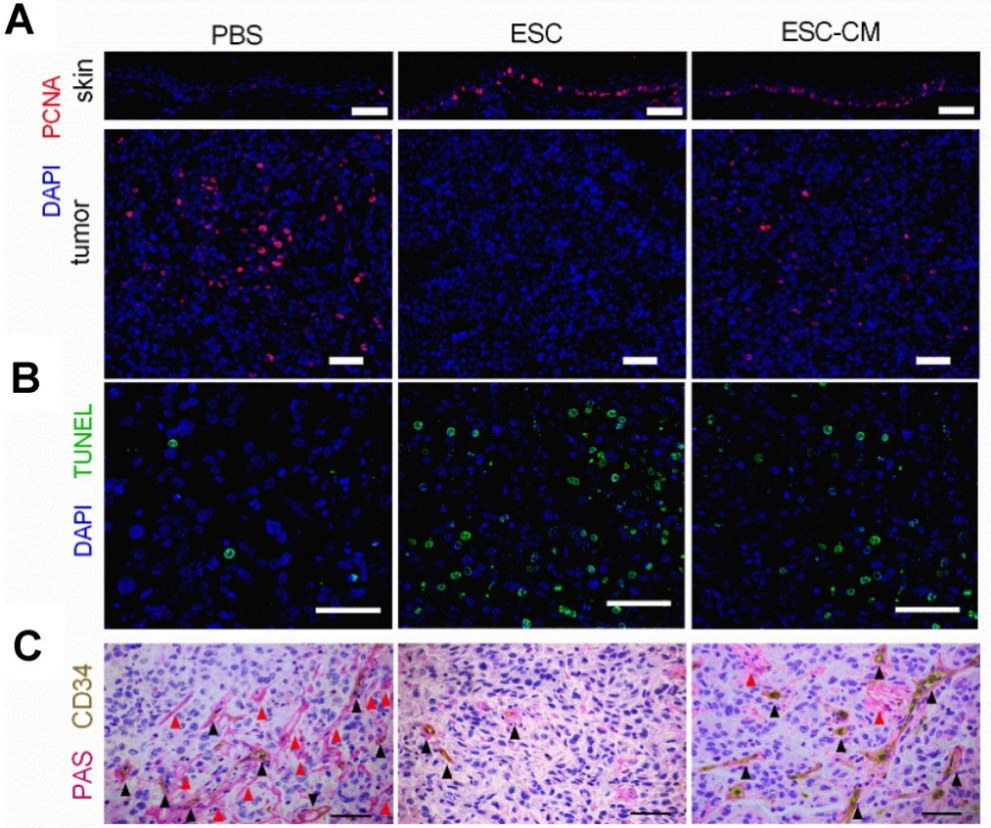
** The ESCMe showed therapeutic effect on apoptosis, proliferation and intratumoral microvascular structures in tumor-bearing mice. exerts powerful anti-tumor activity (A)** Staining of PCNA in tumors and surrounding skin tissues obtained from mice 21 days after treatment with PBS, ESCs, or ESC-CM. **(B,C)** TUNEL **(B)** and PAS-CD34 double staining **(C)** in tumor tissues. Red arrowheads mark VM; black arrowheads mark normal endothelial-dependent vessels. Scale bar, 50 μm. **(A-C)**

To determine whether ESCs can reverse the malignant phenotype of C918 cells and protect the normal cells from senescence *in vivo*, we established a mouse tumor model by subcutaneously injecting C918 cells into nude mice. When the tumor developed to a size of approximately 150 mm^3^ (around two weeks after injection), the mice were randomly divided into three groups and injected with ESCs, ESC- conditioned medium (ESC-CM), or phosphate- buffered saline (PBS) at two different peritumoral sites once in a week for three weeks (Figure S7A). After three treatment cycles, the mice were euthanized, and the tumors and surrounding skin tissues were examined. After 8 d of treatment, the tumors in the ESC-treated mice were much smaller, whereas the tumors in the ESC-CM treated mice were slightly smaller than those in the PBS-treated mice. (Figure 6A-B). Proliferating cell nuclear antigen (PCNA) is an indicator of cell proliferation, and we found significantly fewer PCNA+ cells (Figure 7A) and more apoptotic cells (Figure 7B) in the tumors of mice treated with ESCs than in those of mice treated with PBS or ESC-CM. In addition, the tumors from ESC-treated mice had fewer microvascular structures, including VM and normal endothelial-dependent vessels (Figure 7C), as well as lower N:C ratios (Figure S7B). Furthermore, the tumors of the ESC-treated mice ulcerated at volumes as small as 200 mm^3^ (Figure 6B), whereas the tumors in the PBS-treated group did not ulcerate even at volumes exceeding 2000 mm^3^. We attribute this to an increase in the number of apoptotic cells and a reduction in the number of vessels in the tumors from ESC-treated mice. Indeed, a histopathological assay indicated robust improvements in the liver and spleen lesions of ESC-treated mice (Figure S7C- D). Compared with that in the PBS or ESC-CM treatment groups, the surrounding skin tissue in the ESC treatment group had more PCNA+ cells (Figure 7A), which correlated with the *in vitro* experiments. All of these improvements were blunted to varying degrees by ESC-CM treatment (Figure 6-7, and Figure S7B-D).

**Figure 8:**
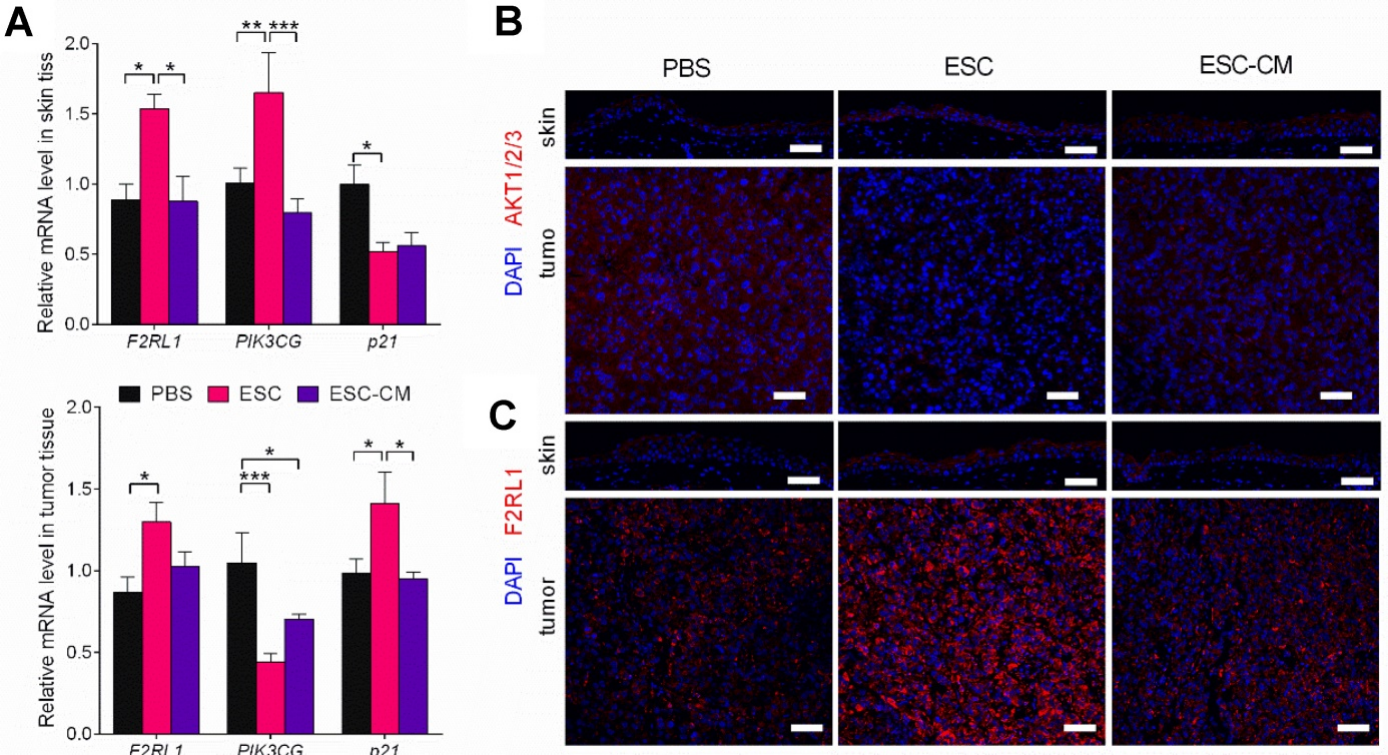
** The ESCMe can regulate PI3K pathway bidirectionally *in vivo*. (A)** *F2RL1*, *PIK3CG*, and *p21* expression in skin and tumor tissues, as assessed by RT-qPCR. Scale bar, 50 μm. Delta CT means of these genes in skin and tumor tissues were shown in Table S6 and Table S7. **(B,C)** Staining of AKT1/2/3 (**B**) and F2RL1 (**C**) in tumors and surrounding skin tissues obtained from mice 21 days after treatment with PBS, ESCs, or ESC-CM. Data are means ± SEMs. *P< 0.05. Scale bar, 50 μm.

To identify the molecular changes in the PI3K pathway *in vivo*, we assessed the expression of PI3K pathway-related genes in tumors and adjacent skin tissues. Following ESC treatment, while the *PIK3CG* gene was downregulated and the *p21*gene was upregulated in tumor tissue, whereas the *PIK3CG* gene was upregulated and the *p21* gene was downregulated in skin tissues (Figure 8A). AKT expression was decreased in tumor tissue but increased in skin tissue (Figure 8B). F2RL1 expression was increased in both tissues transcriptionally and translationally (Figure 8A and C). Indeed, these results were in agreement with the *in vitro* results. Expression levels of the *PIK3CG*, *p21*, and *F2RL1* genes in the tumor and skin tissues from ESC-CM- and PBS-treated mice did not differ significantly (Figure 8A-C). Altogether, these results demonstrated that the ESCMe could suppress the aggressive phenotype of tumor cells while preventing the senescence of normal somatic cells through a bidirectional regulation of the PI3K pathway.

### Suicide gene ensure the safety of ESC application

ESC transplantation poses a risk of teratoma formation 33,34. The incidence of teratoma formation is associated with the dose, site, and time of ESC transplantation 35. To reduce the risk of teratoma formation resulted from ESC transplantation, we transfected ESCs with a suicide gene, herpes simplex virus thymidine kinase (HSV-TK), controlled by ganciclovir (GCV) (Figure S7E). We used GCV to control the lifetime of the ESC-TK *in vivo*. Once the ESCs are differentiated, their ability to maintain the embryonic microenvironment is significantly reduced or completely abolished, which consequently reduces their therapeutic efficacy and then induces differentiated ESCs to form teratomas. Therefore, differentiated ESCs must be eliminated, and freshly prepared undifferentiated ESCs must be added to maintain the embryonic microenvironment. To evaluate the distribution of ESCs *in vivo*, we transplanted ESCs labeled with 1,1'-dioctadecyl-3,3,3',3'- tetramethylindotricarbocyanine iodide (DiR), a near- infrared fluorescent dye, into mice. The *in vivo* live imaging analysis showed that ESCs were clustered at the site of transplantation around the tumor on day 1 (Figure S7F). By day 5, the area showed an enhanced fluorescence intensity, indicating that the ESCs had survived and proliferated *in vivo*. We then injected the mice with GCV intraperitoneally to eliminate the ESCs. Three days later (on day 8), the fluorescent signal was barely detectable, suggesting that the ESCs had been eliminated or substantially reduced. In mice injected with DiR-labeled ESCs-TK that did not receive a subsequent injection of GCV, the fluorescent signals detected on day 8 were stronger than those detected on day 5, indicating that ESCs would continue proliferating *in vivo* without GCV administration. We injected ESCs-TK on day 8 for another cycle of 7 d. No teratomas were detected after three cycles of treatments, which was consistent with our previous observations in a leukemia mouse model 17. We demonstrated that this could be a safe approach to monitor and eliminate ESCs before their differentiation to prevent the formation of teratomas.

## Discussion

The main objective of current anti-cancer therapies is the killing of cancer cells, which inevitably leads to adverse effects on normal somatic cells. Previous studies have demonstrated that the early embryonic microenvironment could reprogram cancer cells towards a benign phenotype 8,9. In the present study, we transplanted ESCs in mice bearing uveal melanoma cancer to recapitulate the early embryonic microenvironment. Our study demonstrated that ESCs could significantly restrict the growth and malignant phenotype of tumors by promoting a higher level of the apoptotic cells as well as fewer proliferating cells, and microvascular structures in the tumor tissues. In a previous study, we employed ESCs to a murine leukemia model and found that the proliferation of leukemic cells decreased and the survival of mice increased after injection of ESCs 17. These findings demonstrate that the ESC transplantation promises a beneficial therapeutic utility in cancer treatment.

The capacity of the early embryonic microenvironment to reverse tumor malignancy decreases as the embryo develops, and the effect is the strongest in the early embryo but almost disappears after birth 10,11. In correlation with these findings, our *in vitro* experiments showed that the ESCMe suppressed the proliferation, invasiveness, and tumorigenicity of C918 cells. Neither MSCs nor differentiated CECs share the ability of ESCs to influence the malignant behavior and gene expression of tumor cells. This suggests that non-embryonic cells cannot reverse tumor aggressiveness. Consequently, we used ESCs transfected with a suicide gene to eliminate differentiated ESCs in a controlled manner and replenished fresh and undifferentiated ESCs to maintain the embryonic microenvironment. Moreover, both teratoma formation and immune rejection as a result of differentiated ESCs were reduced or avoided 36,37.

We demonstrated that the suppressive effects of the ESCMe on the proliferation, invasiveness, and tumorigenicity of C918 cells were much more significant in the cell-to-cell contact co-culture system than in the non-contact one. It may be likely due to the cell-cell interaction, being similar to the embryo microenvironment, as ESCs could exert their effects through the paracrine and autocrine pathways, as well as by direct signal communication via cell-cell contact. In agreement with the *in vitro* experimental results, we observed that ESCs treatment showed a superior therapeutic effect in tumor-bearing mice to the ESC-CM treatment in terms of tumor growth, apoptosis, proliferation, and intratumoral microvascular structures.

The senescence-associated secretory phenotype, which includes a variety of inflammatory factors, growth factors, and proteases secreted by aging cells, creates a cancer-prone microenvironment that favors tumor progression aggressively with aging 38. To recapitulate the tumor microenvironment *in vivo*, we cultured tumor cells and normal somatic cells with ESCs in a contact co-culture system. Our results indicated that the ESCMe could suppress the various malignant behaviors of uveal melanoma cells while preventing the senescence of RPE cells. To further confirm the bidirectional function of the ESCMe (inhibiting the malignant cell proliferation, and senescence of normal cells)* in vivo*, we transplanted ESCs into tumor-bearing mice and found that ESCs could markedly suppress tumor growth and enhance the proliferation of the adjacent skin tissue. Collectively, these results support the notion that ESCMe can exert tumor-inhibiting properties and modulate the aging tumor-promoting environment by suppressing the senescence of normal cells.

Several reports have demonstrated that the frequent activation of the PI3K/AKT pathway plays an extremely crucial role in the high malignancy of uveal melanoma 24,25,39. Our results showed that the ESCMe inhibited the PI3K pathway in C918 cells, which accounted for its anti-cancer effect. ESCs in direct contact with C918 cells (cell-cell contact co-culture group) had a stronger effect in the suppression of the PI3K pathway.

Similarly, *in vivo* experiments also demonstrated that the PI3K pathway related- genes of tumors tissues were changed more significantly in the ESC-treated mice than in the ESC-CM-treated mice. These results suggest that, through cell-cell contact, the effect of ESCMe in inhibition of the PI3K pathway was robust. In addition, the PI3K pathway is also involved in regulating the proliferation and survival of normal somatic cells. However, its activity becomes weaker with aging, resulting in cellular senescence and decreased proliferation 40. Surprisingly, our findings discovered that the ESCMe can downregulate the PI3K pathway in tumor cells but upregulate in somatic cells, thus playing a dual role in reversing the malignancy of the tumor as well as preventing the senescence of somatic cells both *in vitro* and *in vivo*. An intriguing finding is that both mRNA and protein levels of F2RL1 were increased in both the tumor cells and normal cells after ESC treatment. Studies have shown that the activation of the F2RL1/PI3K pathway had anti-apoptotic effects on intestinal epithelial cells and the neutrophils of normal and allergic subjects 31,41. Researchers have also found that PI3K, a downstream effector of F2RL1 activation, has a negative regulatory role in limiting the proinflammatory gene expression induced by F2RL1. Therefore, PI3K may act to minimize the potential negative consequences of the activated inflammatory responses 42. Given that F2RL1 is not only a cell surface receptor but is also related to PI3K signaling in various physiological and pathological processes, such as inflammation and cancer, its upregulation may be a key to the role of the ESCMe. Further experiments are needed to elucidate the potential role of F2RL1 in the ESCMe-mediated bidirectional regulation of the PI3K signaling pathway.

## Conclusions

Our study provides several lines of evidence that ESCs can suppress the malignant phenotype of tumor cells while repressing the senescence of normal cells through the bidirectional regulation of the PI3K pathway. These findings provide new avenues for the development of ESC transplantation for the treatment of cancer through the reversal of the cancer- permissive microenvironment rather than by the killing of tumor cells directly.

## Supplementary Material

Supplementary figures and tables.

## Abbreviations

ESCs: embryonic stem cells
PI3K: phosphoinositide 3-kinase
TK: thymidine kinase
ESCMe: embryonic stem cells microenvironment
CCo group: ESCs in a cell-to-cell contact co-culture system
VE-cadherin: vascular endothelial cadherin
VEGF-A: vascular endothelial growth factor A
FGF2: fibroblast growth factor 2
MMPs: matrix Metalloproteinases
BRMS1: breast cancer metastasis suppressor 1
TXNIP: thioredoxin-interacting protein
TUNEL: TdT-mediated dUTP Nick-End Labeling
PDK2: pyruvate dehydrogenase kinase isoform 2
mTOR: mechanistic target of rapamycin
CD34-PAS: CD34 endothelial marker periodic acid-Schiff dual staining
VM: Vascular mimicry
TCo group: transwell (non-cell-to-cell contact) co-culture system
MSCs: mesenchymal stem cells
CECs: corneal epithelial cells
RT-qPCR: reverse transcription polymerase chain reaction
RPE: retinal pigment epithelial
KLF4: Kruppel-like factor 4
F2RL1: F2R-like trypsin receptor 1
AKT: protein kinase B
ESC-CM: ESC-conditioned medium
PBS: phosphate-buffered saline
PCNA: proliferating cell nuclear antigen
HSV-TK: herpes simplex virus thymidine kinase
ESC-TK: ESCs with a suicide gene
GCV: ganciclovir
DiR: 1,1'-dioctadecyl-3,3,3',3'-tetramethylindotricarbocyanine iodide
SASP: senescence-associated secretory phenotype
DiO: 3,3'-dioctadecyloxacarbocyanine perchlorate
DiI: 1,1'-dioctadecyl-3,3,3',3'-tetramethylindocarbocyanine perchlorate
