# Supplementary material for: Embryonic Stem Cells Modulate the Cancer-Permissive Microenvironment of Human Uveal Melanoma
Source: Theranostics. 2019 Jul 9;9(16):4764–78. doi: 10.7150/thno.33139 (PMC6643444; doi:10.7150/thno.33139)
Supplement: Supplementary file 1 — Supplementary figures and tables. [file thnov09p4764s1.pdf]

Supplemental Material

Table S1. Primer sequences

Primer	Forward	Reverse
Human-GAPDH	CGTATTGGGCGCCTGGTCAC	ATGATGACCCTTTTGGCTCC
Human-cyclin A2	CGCTGGCGGTACTGAAGTC	GAGGAACGGTGACATGCTCAT
Human-KLF4	TCTCAAGGCACACCTGCGAA	TAGTGCCTGGTCAGTTCATC
Human-p21	TGTCCGTCAGAACCCATGC	AAAGTCGAAGTTCCATCGCTC
Human-p27	TAATTGGGGCTCCGGCTAACT	TGCAGGTCGCTTCCTTATTCC
Human-F2RL1	TTGTGTTTGTGGTGGGTTTGC	ACCAGATGACAGAGAGGAGGT
Human-PDK2	CTTCAGCAAGTTCTCCCCGTC	TCGGGAAGCAGGTTGATCTCT
Human-TXNIP	GCCAGCCAACTCAAGAGACAA	TTCGAGCAGAGACAGACACCC
Human-BRMS1	GCCTCCAAGCAAAGACACAGA	AGCTCTCCTCTTCTGACTCTGT
Human-PDK1	AGAGGGTTACGGGACAGATGC	GTCTTTGGGTTCTCTGCTGGG
Human-CD44	ATGGGTTCATAGAAGGGCACG	TGTCATACTGGGAGGTGTTGGA
Human-RhoA	TTGTTGGTGATGGAGCCTGTG	GCCAACTCTACCTGCTTTCCA
Human-GAB1	CAGACAGCGAGGAGGAGATGA	GGTGGATGGTGGTGCTGTATT
Human-PTEN	CTCAGCCGTTACCTGTGTGTG	AGGTTTCCTCTGGTCCTGGTA
Human-AKT2	AGGCACGGGCTAAAGTGAC	CTGTGTGAGCGACTTCATCCT
Human-AKT3	GGAGAGGAAGAGATGGATGCCT	CCACTTGCCTTCTCTCGAACC
Human-mTOR	CCCTCCATCCACCTCATCAGT	CGCCAAGACACAGTAGCGAAT
Human-PIK3CG	AACACCGACCTCACAGTTTTT	CTCAAGCCACACATTCCACAG
Human-cyclin B1	GGTTGTTGCAGGAGACCATGT	AACATGGCAGTGACACCAACC
Human-cyclin D1	TTCATTTCCAATCCGCCCTCC	TGTGAGGCGGTAGTAGGACAG
Human-FGF2	AGAAGAGCGACCCTCACATCA	CGGTTAGCACACACTCCTTTG
Human-MMP1	AAAATTACACGCCAGATTTGCC	GGTGTGACATTACTCCAGAGTTG
Human-MMP9	AGACCTGGGCAGATTCCAAAC	CGGCAAGTCTTCCGAGTAGT
Human-VEGF-A	CCTCCGAAACCATGAACTTT	CCACTTCGTGATGATTCTGC
Human-VEGF-B	GATGGCCTGGAGTGTGTG	CACACTGGCTGTGTTCTTCC
Human-VE-cadherin	TGTTCACGCATCGGTTGTTCA	TACATGACAGAGGCGTGGTCT

Human- VEGF-C	GGCTGGCAACATAACAGAGA	GTGGCATGCATTGAGTCTTT
Human- FAK	GTTATCCCAGTCCGAGGTCCA	TGACCTGGATAGATGCTGCCA
Mouse- Gapdh	AGTGTTTCCTCGTCCCGTAGA	TGAGGTCAATGAAGGGGTCGT
Mouse- F2rl1	AGTTCTCTGCGTCCATCCTCA	GCGGGGTGTTTCTTCTTCGTT
Mouse- Pik3cg	TGCGGAGTTCTACCACCGAT	TAATGCAGGCAGTCTAGGGTC
Mouse-p21	TCTTCCCCATCTTCGGCCTTA	AGTTTGGAGACTGGGAGAGGG

Table S2. Antibodies, source and dilution

Antibody	Source	Dilution(application)
AKT1/2/3	Abcam#ab131443	1:10000(WB）<br>1:100(IF)
p27	Abcam#ab32034	1:1000 (WB）<br>1:100 (IF)
MMP1	Abcam#ab38929	1:1000(IF)）
mTOR	Abcam#ab2732	1:2000(WB）<br>1:100(IF)
PTEN	Abcam#ab32199	1:10000(IF)）
cyclinA2	Abcam#32386	1:10000 (WB）<br>1:200(IF)
p21	Abcam#109520	1:2000（WB）<br>1:1000(IF)
F2RL1	Abcam#180953	1:2000 (WB）<br>1:100(IF)
cyclinB1	Abcam#181593	1:2000 (WB）<br>1:500(IF)
PDK2	Novusbio#NBPI-87307	1:200(WB）<br>1:100(IF)
KLF4	Abcam#215036	1:10000(WB）
VEGF-A	Abcam#46154	1:100(WB）
cyclinD1	Abcam#134175	1:10000(WB）<br>1:50(IF)
FGF2	Abcam#8880	1:1000(WB)<br>1:200(IF)
TXNIP	Abcam#188865	1:1000(WB)
BRMS1	Abcam#57082	1:100(WB）
β-actin	SIGMA#A5441	1:3000(WB)
MMP9	Abcam#38929	1:250(IF)

VE-cadherin	Abcam#33168	1:100(IF)
CD34	Abcam#8158	1:50(IF)
PCNA	Cell Signaling#D3H8P	1:800(IF)
Alexa Fluor 594 donkey anti-rabbit IgG	Molecular Probes#A21207	1:1000(IF)

Table S3. Delta CT means of genes in C918 cells with VO

	Ctrl-T			TCo			TCo+VO			Ctrl-C			CCo			CCo+VO		
cyclinA2	7.60	7.34	7.37	7.41	7.64	7.63	7.11	7.01	7.06	8.07	7.70	8.68	7.68	7.30	8.16	7.60	7.34	7.37
cyclinB1	5.97	5.74	6.01	6.63	6.42	6.97	6.19	5.72	5.59	6.10	6.29	6.02	5.80	5.84	5.95	5.86	5.98	5.69
cyclinD1	6.01	5.77	4.87	7.74	7.12	6.41	6.14	5.86	5.89	5.97	7.08	6.40	5.00	5.90	5.38	4.92	6.35	5.83
FGF2	9.68	9.02	9.68	10.07	9.20	9.92	10.01	9.01	9.41	10.46	9.34	10.23	9.93	9.08	10.16	9.68	9.02	9.59
MMP1	14.97	14.97	14.97	15.43	15.20	15.89	14.54	14.30	14.49	16.01	16.08	15.83	15.98	15.74	15.38	14.97	15.44	14.97
MMP9	13.60	14.93	14.87	14.92	16.00	15.80	14.30	16.12	16.32	15.99	14.90	16.23	16.57	15.17	16.31	14.93	13.60	14.25
VEGF-A	8.70	10.90	8.35	9.23	11.40	8.95	8.25	10.32	8.23	9.43	8.49	10.49	7.96	5.99	8.20	8.08	6.55	8.35
VEGF-B	0.18	7.19	7.64	0.76	8.05	8.44	-0.05	7.04	7.16	-0.23	7.43	8.52	-0.78	7.05	8.04	0.18	8.03	9.24
VE-cadherin	8.60	9.81	9.46	9.39	10.15	10.45	9.13	10.02	10.28	11.28	10.29	10.64	10.59	10.09	10.16	9.81	9.00	8.60
p21	5.18	5.03	4.96	4.85	5.01	4.27	5.21	6.09	4.85	5.04	4.27	4.62	5.20	4.70	5.07	5.18	5.03	4.96
p27	8.28	8.22	8.24	7.50	7.76	7.74	7.66	7.83	8.04	7.76	7.77	7.62	7.99	7.70	7.78	8.28	8.22	8.24
PIK3CG	10.62	12.21	11.25	11.77	13.18	11.99	11.36	13.04	11.66	10.99	9.87	9.95	11.52	10.61	11.04	11.77	11.17	10.92
PDK1	9.33	11.27	8.87	9.80	12.04	9.05	9.49	11.15	8.43	8.87	9.44	9.78	9.17	9.89	10.27	8.98	9.50	9.83
PDK2	9.59	8.98	10.01	10.05	9.67	10.45	9.78	9.23	9.90	9.59	8.98	9.58	10.82	10.09	10.47	9.97	9.41	10.11
AKT2	19.53	20.88	19.79	20.02	21.09	20.50	18.87	20.08	18.93	18.87	18.28	19.79	19.75	20.30	20.97	19.33	18.89	20.34
AKT3	9.16	9.02	9.50	9.54	9.54	9.68	8.84	8.82	9.54	9.16	8.27	9.76	9.75	8.92	10.13	9.05	8.53	9.65
mTOR	8.77	8.15	8.95	9.84	9.66	9.39	9.01	8.34	8.67	8.95	9.27	8.86	9.32	10.12	9.24	8.79	9.16	8.93

Table S4. Delta CT means of genes in C918 cells

	Ctrl			TCo			CCo		
CD44	-0.75	3.74	4.03	0.32	5.38	5.71	-0.38	4.02	4.47
RhoA	4.62	4.28	4.45	4.52	3.96	3.97	5.16	4.62	5.16
GAB1	10.92	11.01	11.38	10.23	10.18	10.92	12.16	11.81	12.49
PTEN	8.07	0.79	1.47	7.09	-0.10	0.93	7.73	0.43	1.30

Table S5. Delta CT means of genes in RPE and C918 cells

	RPE-Ctrl			RPE-ESC			C918-Ctrl			C918-ESC		
PAR2	11.32	13.90	5.57	9.94	11.43	3.55	15.06	12.64	9.63	12.70	10.01	7.66
PIK3CG	13.05	13.05	4.56	12.00	12.35	3.98	6.53	10.94	6.53	7.37	11.52	6.86
PDK1	10.20	8.63	8.99	8.88	7.09	8.43	7.93	7.93	4.07	8.62	9.04	8.16
PDK2	8.84	2.55	7.87	8.00	1.92	7.48	9.59	9.59	8.08	9.64	9.77	9.02
AKT2	19.89	20.73	13.52	19.55	20.16	12.56	14.34	14.54	20.03	14.54	14.70	20.27
AKT3	8.67	8.67	2.27	7.99	8.26	1.81	2.07	9.02	7.50	2.51	9.44	8.75
mTOR	2.96	2.54	2.54	2.75	2.02	2.33	8.66	8.82	8.93	9.74	9.47	9.33
PTEN	6.13	5.25	6.95	6.47	6.01	7.23	7.96	7.96	7.75	7.45	7.75	7.65
cyclin A2	8.01	8.49	9.01	7.84	7.25	7.33	6.94	8.02	6.78	7.31	8.79	8.04
cyclin B1	6.82	5.97	7.00	6.70	5.98	5.42	4.02	3.79	4.99	4.71	4.97	5.94
cyclin D1	5.29	5.08	5.45	4.95	3.95	4.18	4.60	3.33	4.84	5.97	4.94	6.38
p21	4.41	6.00	5.27	5.99	6.61	6.11	5.98	6.43	5.98	5.32	5.44	5.61
p27	7.58	7.51	6.96	8.05	8.17	7.81	9.68	9.36	9.93	9.32	9.04	9.97
KLF4	9.01	12.61	10.99	7.22	10.22	8.94	6.97	6.66	9.07	8.87	10.40	9.83

Table S6. Delta CT means of genes in skin tissues

	PBS						ESC								ESC-CM				
F2RL1	4.91	4.82	4.15	4.35			3.9	3.76	3.56						4.05	4.84	4.91		
PIK3CG	7.16	7.82	7.44	8.25	7.17	8.02	6.17	7.55	7.04	7.22	6.79				7.49	7.63	8.6	8.15	8.02
p21	0.68	0.4	0.41	0.35			0.83	1.61	0.97	0.91	1.74	0.4	0.11	0.43	0.79	1.03	0.3	1.48	

Table S7. Delta CT means of genes in tumor tissues

	PBS						ESC					ESC-CM						
F2RL1	10	10.56	9.98	10.11	11.06	10.03	9.32	9.8	9.72	9.89		10.05	9.7	9.8	10.4	10.15		
PIK3CG	10.15	10.14	11.21	10.71	11.43		12.57	11.76	11.65	12.11	11.57	11.36	11	11.28	11.02	11.26	11.42	11.21
p21	5.13	5.64	5.22	5.4			4.61	4.88	5.3			5.33	5.59	5.44	5.57			

Figure S1

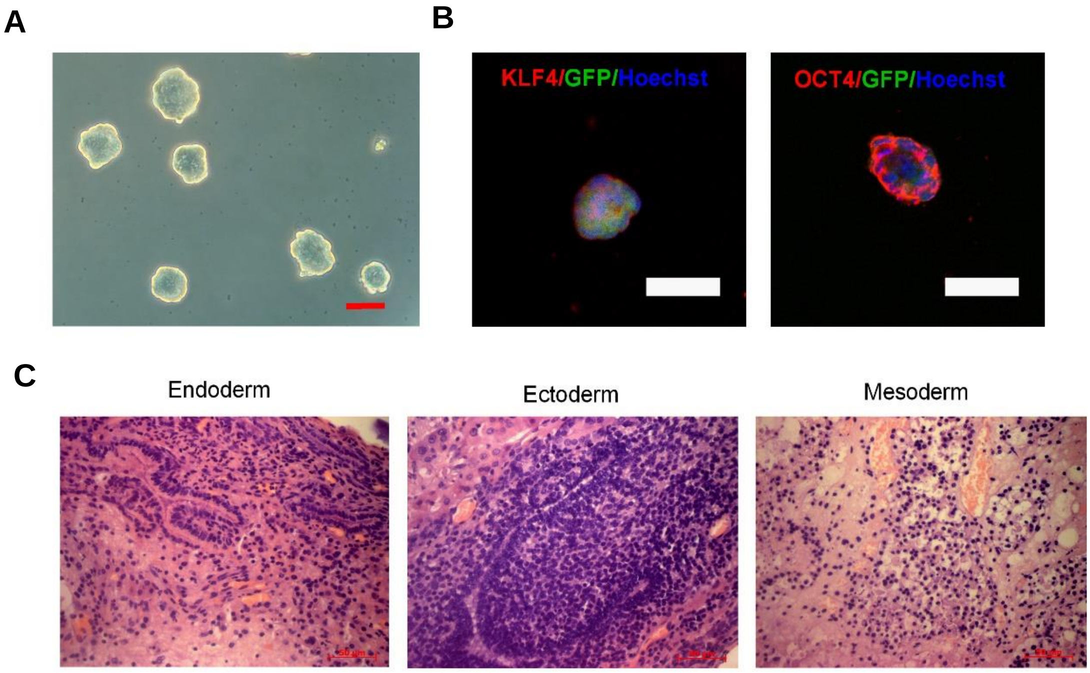

Figure S1. Characteristics of ESCs. (A) Representative images of morphology by phase microscopy. **(B)** The expression of KLF4, OCT4 and GFP as determined by immunofluorescent staining. **(C)** Teratoma formed by ESCs stained with hematoxylin and eosin for histological analysis. Scale bar, 50 µm. (**A-C**)

Figure S2

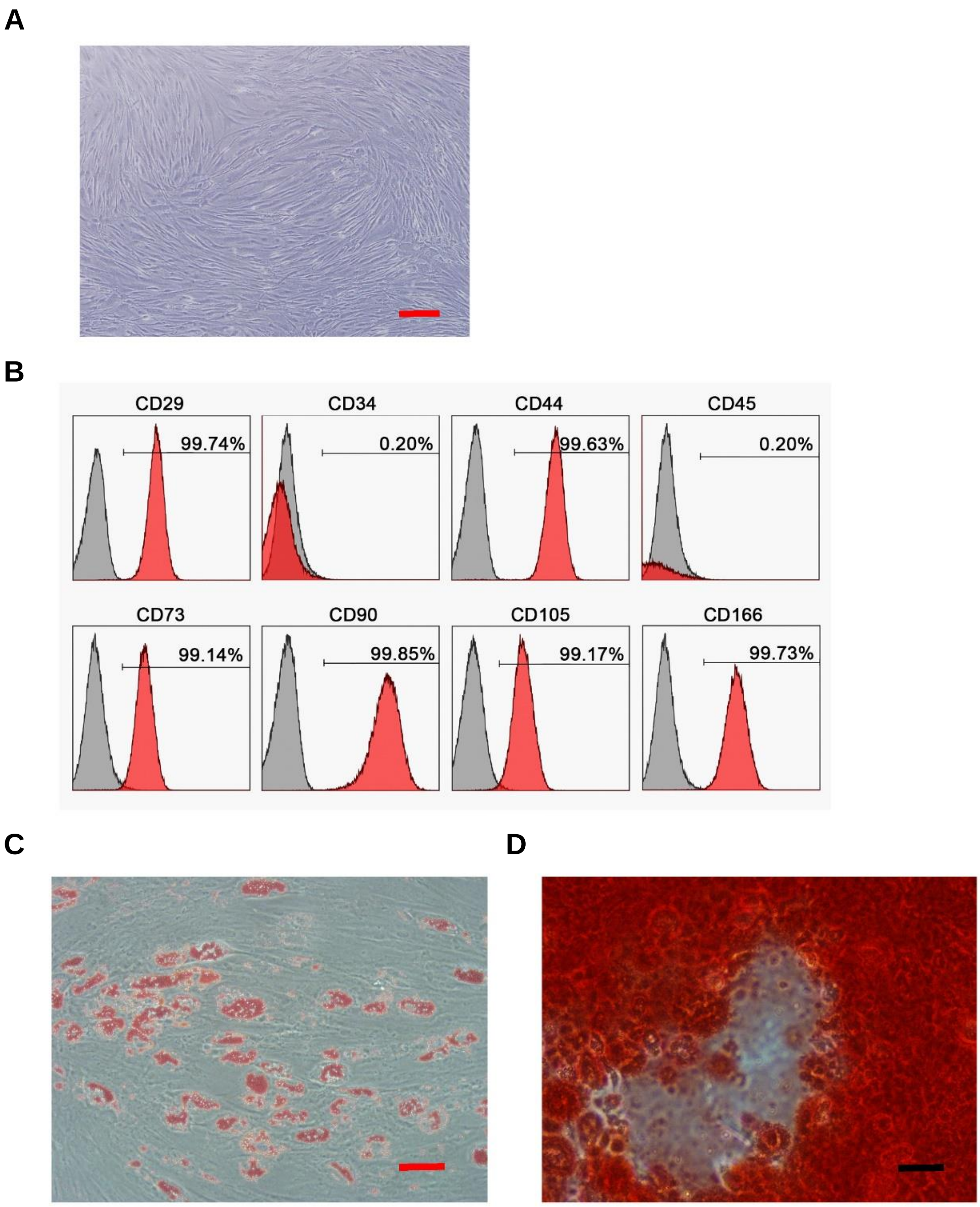

Figure S2. Characteristics of MSCs. (A) Representative images of morphology by phase microscopy. Scale bar, 100 μm. **(B)** Flow cytometry analysis of cell surface markers of MSCs. **(C)** Oil red O staining showing

adipogenic differentiation of MSCs. **(D)** Alizarin red S staining showing osteogenic differentiation of MSCs. Scale bar, 50 μm. (**C-D**)

Figure S3

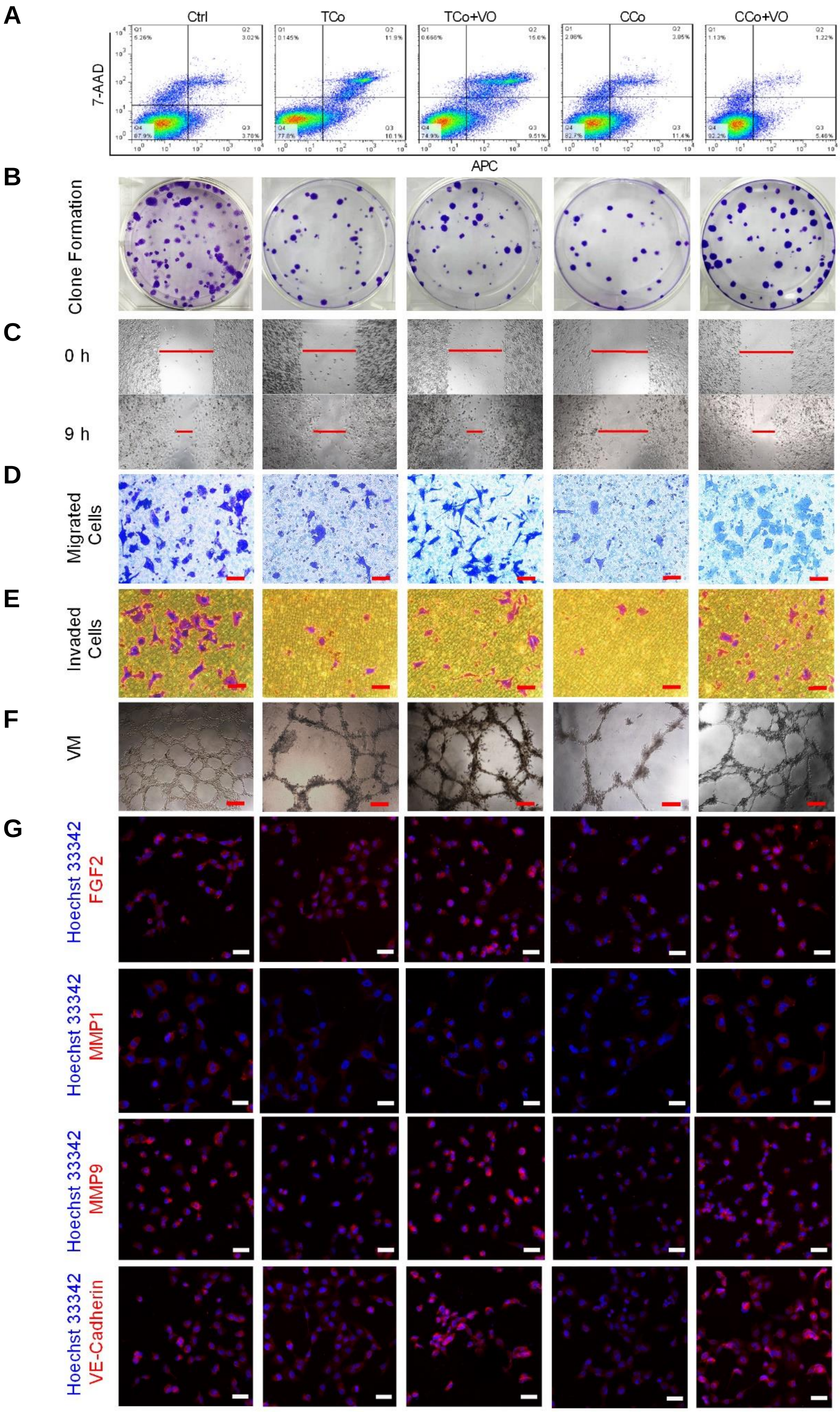

A
Ctrl
TCo
TCo+VO
CCo
CCo+VO
7-AAD
APC
B
Clone Formation
C
0 h
9 h
D
Migrated Cells
E
Invaded Cells
F
VM
G
Hoechst 33342 FGF2
Hoechst 33342 MMP1
Hoechst 33342 MMP9
Hoechst 33342 VE-Cadherin

Figure S3. The ESCMe significantly inhibits the growth and metastasis capacity of C918 cells. (A) Scatter diagram of the proportions of apoptotic C918 cells sorted from Ctrl, TCo, TCo+VO, CCo, and CCo+VO groups, as assessed by flow cytometry. **B-F**, Representative images of clone formation (**B**), wound healing at 0 and 9 h (**C**), migration (**D**), invasion (**E**), and VM (**F**) of C918 cells sorted from Ctrl, TCo, TCo+VO, CCo, and CCo+VO groups. (**G)**, Immunofluorescence assays of FGF2, MMP1, MMP9, and VE-cadherin in C918 cells sorted from Ctrl, TCo, TCo+VO, CCo, and CCo+VO groups. Scale bars: 100 μm (**D-E**), 400 μm (**F**), and 50 μm (**G**).

Figure S4

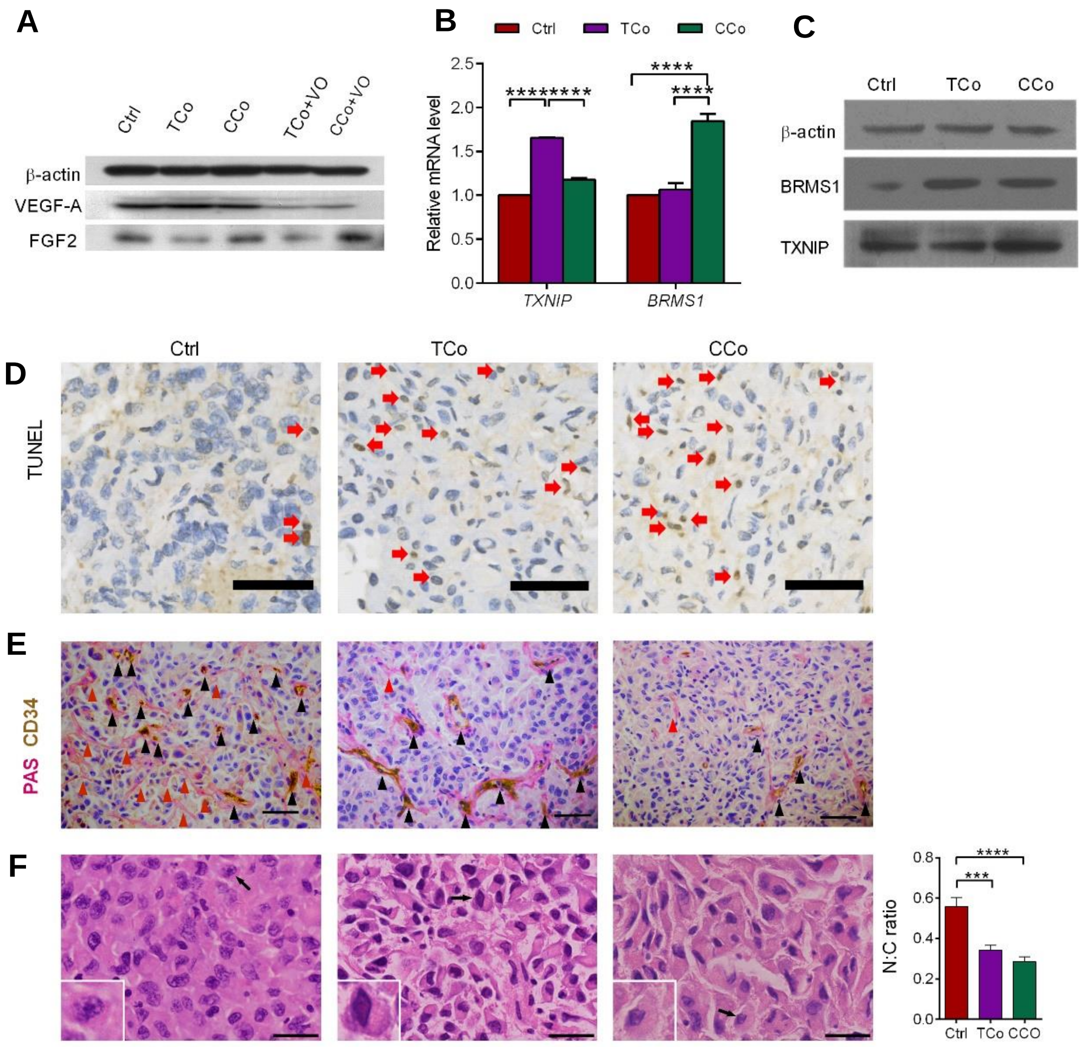

Figure S4. C918 cells treated with ESCs have reduced malignancy and tumorigenesis. (A) Western blotting of VEGF-A and FGF2 in C918 cells. β-actin served as the internal control. **B-C**, RT-qPCR (**B**) and Western blot analyses (**C**) of TXNIP and BRMS1 in C918 cells sorted from Ctrl, TCo, and CCo groups (n = 3 biological repeats). β-actin served as the internal control. **(D)** TUNEL assay of tumor tissues obtained from mice subcutaneously injected with C918 cells sorted from Ctrl, TCo, or CCo groups. Arrows mark

apoptotic cells. (**E)**, PAS-CD34 staining of tumor tissues obtained from mice subcutaneously injected with C918 cells sorted from Ctrl, TCo, or CCo groups. Red arrowheads mark VM; black arrowheads mark normal endothelial-dependent vessels. (**F)** H&E staining of tumor tissues obtained from mice subcutaneously injected with C918 cells sorted from Ctrl, TCo, or CCo groups and N:C ratio analysis. . Scale bar, 50 μm. (**D-F**) Data are means ± SEMs. **P< 0.01; ***P< 0.001; ****P< 0.0001.

Figure S5

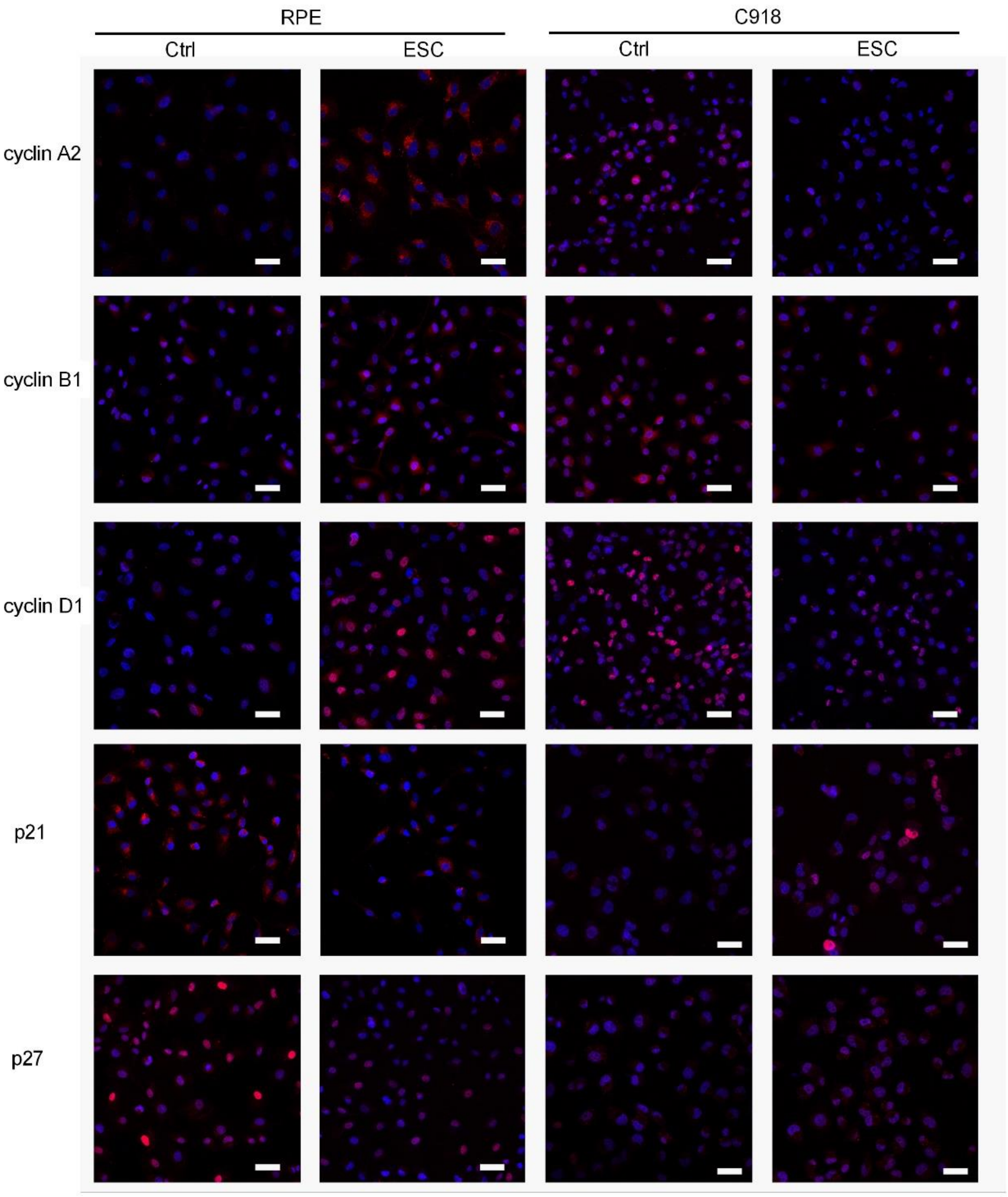

Figure S5. The ESCMe influences the expression of cell cycle promoters and senescence markers. Immunofluorescence staining of cell cycle promoters (cyclin A2, cyclin B1, cyclin D1) and senescence markers (p21, and

p27) in RPE and C918 cells sorted from the Ctrl group (C918 cells co-cultured with RPE cells) and ESC group (C918 cells co-cultured with RPE cells and ESCs). Scale bar, 50 μm.

Figure S6

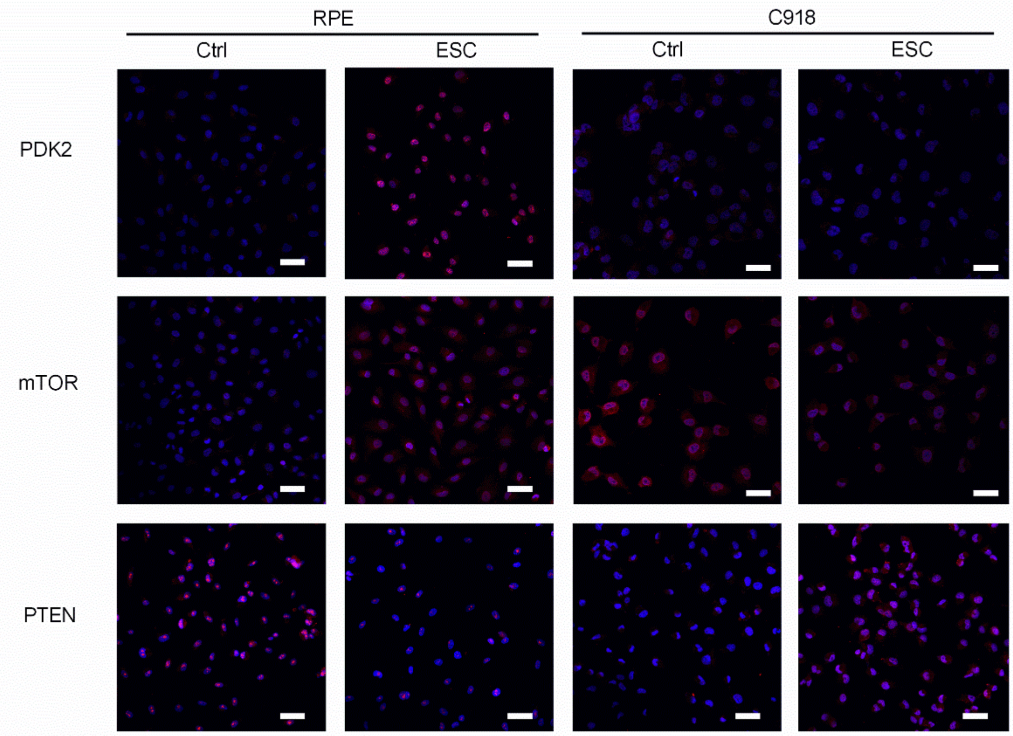

Figure S6. ESC treatment alters the expression of PI3K pathway factors in RPE cells and C918 cells. Immunofluorescence staining of PDK2, MTOR, and PTEN in RPE and C918 cells sorted from the Ctrl and ESC groups. Scale bar, 50 μm.

Figure S7

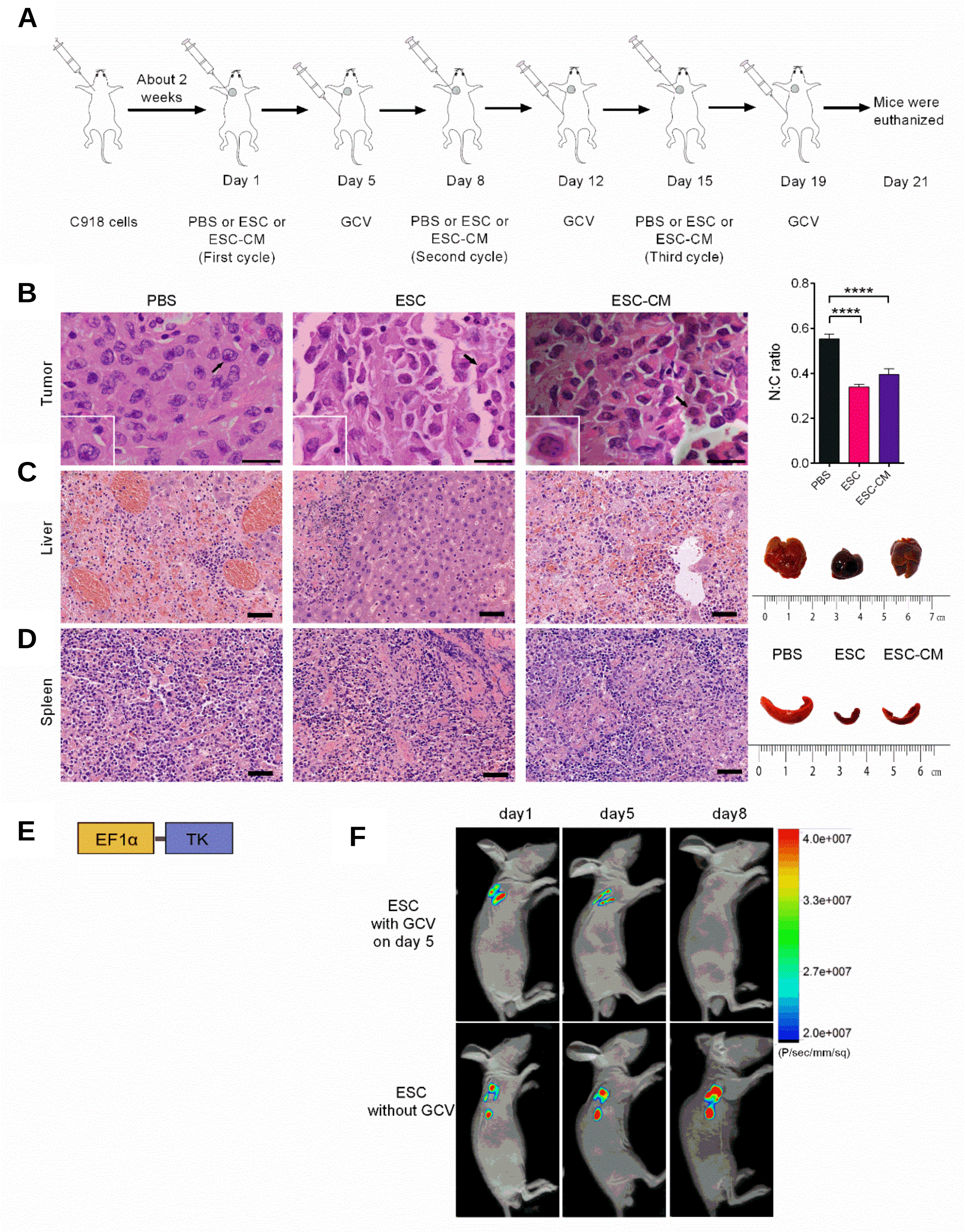

A
About 2 weeks
Mice were euthanized
Day 1
Day 5
Day 8
Day 12
Day 15
Day 19
Day 21
C918 cells
PBS or ESC or ESC-CM (First cycle)
GCV
PBS or ESC or ESC-CM (Second cycle)
GCV
PBS or ESC or ESC-CM (Third cycle)
GCV
B
PBS
ESC
ESC-CM
Tumor
N:C ratio
0.8
0.6
0.4
0.2
0.0
****
****
PBS
ESC
ESC-CM
C
Liver
D
Spleen
PBS
ESC
ESC-CM
E
EF1α
TK
F
day1
day5
day8
ESC with GCV on day 5
ESC without GCV
4.0e+007
3.3e+007
2.7e+007
2.0e+007
(P/sec/mm/sq)

Figure S7. The ESCMe suppresses C918 tumor growth in xenograft mouse models. (A) Schematic workflow of the *in vivo* experiment. Mice were subcutaneously injected with C918 cells and then treated with PBS, ESCs, or ESC-CM. (**B)** H&E staining of tumor tissue and N:C ratio analysis. (**C)-(D)**, Representative images of H&E staining in the livers and spleens of mice treated with PBS, ESCs, or ESC-CM. (**E)** Sketch map of the TK suicide gene. (**F)** In vivo imaging of ESCs dyed with DiR. Scale bar, 50 μm. (**B-D**) Data are means ± SEMs. *P< 0.05; **P< 0.01; ***P< 0.001; ****P< 0.0001.
